# Ulcerative Colitis Seems to Imply Oral Microbiome Dysbiosis

**DOI:** 10.3390/cimb44040103

**Published:** 2022-03-30

**Authors:** Natalia Molinero, Diego Taladrid, Irene Zorraquín-Peña, Miguel de Celis, Ignacio Belda, Alex Mira, Begoña Bartolomé, M. Victoria Moreno-Arribas

**Affiliations:** 1 Institute of Food Science Research (CIAL), CSIC-UAM, Campus de Cantoblanco, Nicolás Cabrera 9, 28049 Madrid, Spain; natalia.molinero@csic.es (N.M.); d.taladrid@csic.es (D.T.); irene.zorraquin@csic.es (I.Z.-P.); b.bartolome@csic.es (B.B.); 2 Department of Genetics, Physiology and Microbiology, Complutense University of Madrid, 28040 Madrid, Spain; migueldecelis@ucm.es (M.d.C.); ignaciobelda@ucm.es (I.B.); 3 Center for Advanced Research in Public Health, Department of Health and Genomics, FISABIO Foundation, 46020 Valencia, Spain; mira_ale@gva.es

**Keywords:** ulcerative colitis, oral microbiome, dysbiosis, differential abundance, alpha diversity, oral biomarkers

## Abstract

Ulcerative colitis (UC) is a recurrent pathology of complex etiology that has been occasionally associated with oral lesions, but the overall composition of the oral microbiome in UC patients and its role in the pathogenesis of the disease are still poorly understood. In this study, the oral microbiome of UC patients and healthy individuals was compared to ascertain the possible changes in the oral microbial communities associated with UC. For this, the salivary microbiota of 10 patients diagnosed with an active phase of UC and 11 healthy controls was analyzed by 16S rRNA gene sequencing (trial ref. ISRCTN39987). Metataxonomic analysis revealed a decrease in the alpha diversity and an imbalance in the relative proportions of some key members of the oral core microbiome in UC patients. Additionally, *Staphylococcus* members and four differential species or phylotypes were only present in UC patients, not being detected in healthy subjects. This study provides a global snapshot of the existence of oral dysbiosis associated with UC, and the possible presence of potential oral biomarkers.

## 1. Introduction

Inflammatory bowel diseases (IBDs) represent a group of chronic, idiopathic, relapsing disorders of unclear etiology affecting millions of people worldwide. IBDs are caused by an inappropriate and persistent inflammatory response to commensal gut microbiota and other environmental factors in genetically susceptible individuals, leading to a deterioration of the gastrointestinal structure and function [1,2,3]. IBDs are categorized into two clinical types: Crohn’s disease (CD), and ulcerative colitis (UC), the latter being characterized by continuous, diffuse, and superficial mucosal inflammation that starts in the rectum and extends to proximal segments of the colon [3]. Several studies have been focused on characterizing the dysbiotic state present in the gut microbiome of UC patients, reporting a decrease in bacterial diversity accompanied by a decrease in relative proportions of *Enterococcus* and *Bacteroides*, and in members of *Clostridium* subcluster XIVa, as well as increases in several opportunistic pathogens [4]. However, IBDs—both CD and UC—have also been associated with several extraintestinal manifestations, such as oral lesions, which can occur either concomitantly with intestinal symptoms or before the presentation of disease, and may even be the primary presenting sign in some cases [5,6]. Oral lesions are more common in CD compared to UC, and are more prevalent in children compared to adults [7]. The main oral manifestations of IBD are cobble-stoning of the oral mucosa, labial swellings with vertical fissures, pyostomatitis vegetans, angular cheilitis, perioral erythema, and glossitis, with pyostomatitis vegetans being the only condition that is more prevalent in UC patients [7]. Although the oral mucosa lesions and oral symptoms can be more severe during the disease activity period, up to 30% of affected patients continue suffering active oral manifestations despite remission [5,8]. However, despite the existence of different oral manifestations in patients with IBD, the pathogenesis of the disease remains unclear. It has been proposed that specific oral manifestations could result from the direct extension of intestinal inflammation—a hypothesis supported by the elevated levels of proinflammatory cytokines present in the saliva of IBD patients [8]. This general inflammatory state associated with the IBD condition could affect the oral mucosa and lead to an oral dysbiosis that could contribute to the worsening of the inflammatory state and play a crucial role in the oral manifestations of these patients [5]. However, only a few studies have focused on analyzing changes in the oral microbiome of IBD patients—most of them performed in the CD phenotype [9,10,11,12,13,14,15]. Regarding UC, most of the data come from pediatric populations or animal models [2,3,16,17,18], and only one study has focused on the analysis of the oral microbiome of a specific UC cohort, performed in a murine model of colitis [16]. Hence, we still lack a comprehensive understanding of the oral microbiota composition in UC patients in particular, and of the possible correlation between oral dysbiosis and the pathogenesis of UC.

In this context, the aim of this work was to study and characterize the oral microbiome of a selected group of UC patients in an active phase of the disease, and to compare their profiles with those of similar unaffected populations, in order to determine whether—as found in the gut—bacterial dysbiosis also exists in the oral cavity. We also aimed to determine the presence of potential oral microbial biomarkers associated with the onset of the disease. Our findings should provide insight into the association between the human microbiome and UC, and the development of new precautionary or diagnostic methods for patients with UC.

## 2. Material and Methods

### 2.1. Study Design

The study lasted from April 2018 to October 2019, and involved UC patients and healthy donors. UC patients were selected from the patient cohort of the Digestive System Department of the “Infanta Sofia Hospital” (Madrid, Spain) (ISRCTN39987), whereas healthy donors were recruited in the Primary Care Centre “Paseo de la Chopera” (Madrid, Spain) (ISRCTN13100451). As inclusion criteria for patients with UC, participants had no oral manifestations at the time of sampling, did not take any medication that could alter the results obtained, and were in an active phase of the disease. As exclusion criteria for both groups, participants could not have received antibiotics for at least 6 months before the study, suffer from type I diabetes, have severe cardiac, endocrine, or gastrointestinal disorders, have a previous history of alcohol or drug abuse, or follow exclusive diets (e.g., vegan or vegetarian). The study protocol was approved by the Ethics Committee of the Hospital Infanta Sofia and Hospital La Paz (Madrid, Spain) and the Bioethics Committee of The Spanish National Research Council (CSIC), and it was conducted according to the guidelines laid down in the Declaration of Helsinki. All participants were informed about the study, and they signed written informed consent.

For all participants, serum biochemical parameters were measured in plasma using an automated biochemical autoanalyzer. The blood tests included the measurement of glucose levels and lipid profiles, among others. For UC patients, fecal calprotectin was determined by quantitative enzyme-linked immunosorbent assay (ELISA) immediately after fecal sample collection. To analyze the oral microbiome from a global point of view (tongue, teeth, palate, mucosa, etc.), saliva samples were collected after awakening, and after brushing teeth with water (without using toothpaste), but before drinking or eating any type of food. Unstimulated saliva (3–5 mL) was expectorated naturally by drooling—that is, without forcing its production. All of the saliva samples were kept in an ice bath during collection, and then were kept at −80 °C until analysis.

### 2.2. DNA Extraction and Sequencing

Saliva samples were used for DNA extraction using the MasterPure™ Complete DNA and RNA Purification Kit (Epicentre, Madison, WI, USA), following the manufacturer’s instructions, with a prior lysis with lysostaphin from *Staphylococcus staphylolyticus* (5000 U/mL), mutanolysin from *Streptomyces globisporus* ATCC 21553 (2500 U/mL), and lysozyme from chicken egg white (50,000 U/mL) (Sigma-Aldrich, San Luis, MO, USA). The V3–V4 region of the 16S ribosomal RNA gene was amplified using the primers used by Klindworth et al., which produce a PCR product of 460 bp [19]. Primer sequences were as follows: 5′-CCTACGGGNBGCASCAG-3′ and reverse 5′-GACTACNVGGGTATCTAATCC-3′. The two-step Illumina^®^ PCR protocol was followed to prepare the libraries, and samples were submitted to 2 × 300 bp paired-end sequencing by means of an Illumina^®^ MiSeq instrument (Illumina^®^, San Diego, CA, USA). Raw files are available in the National Center for Biotechnology (NCBI) repository under the project code PRJNA749643.

### 2.3. Sequence Processing

To process raw reads (FASTQ files) from the Illumina^®^ instrument, RStudio software v. 4.03 was used. The FASTQC files were filtered for reads with low quality and the presence of alien DNA using DADA2 (v.1.18.0). The DADA2 algorithm was also employed to denoise, join paired-end reads, and filter out chimeras in the raw data [20,21]. This algorithm can allow the differentiation of a single nucleotide, leading to the formation of amplicon sequence variants (ASVs). The taxonomic assignment was performed using the naïve Bayesian classifier implemented in DADA2, using Silva v.138 as a reference database [22], with a bootstrap cutoff of 80%. A total of 1,283,321 complete good-quality reads were used for the analysis. All of the information about the sequencing process, as well as error rates and rarefaction curves to assess sequencing depth, are available in Table S4 and Figures S1 and S2, respectively.

### 2.4. Statistical Analysis

Statistical analyses were conducted in the RStudio environment. Bacterial diversity, expressed in terms of alpha diversity, was estimated using the ASVs by calculating the observed, Shannon, and Simpson indices through the “phyloseq” package. ANOVA analysis was employed to detect significant differences between alpha diversity values. Compositional differences between samples (beta diversity) were obtained by employing a Bray–Curtis dissimilarity matrix represented by non-metric multidimensional scaling (NMDS) through the “vegan” package. Permutational multivariate analysis (PERMANOVA) belonging to the “vegan” package was conducted to find statistical differences between experimental groups, using Holm’s multiple comparison testing correction by default. Differences in the relative abundances of taxa and ASVs were assessed using the Mann–Whitney U test, and subsequently applying Benjamini–Hochberg post hoc correction, with a false discovery rate (FDR) threshold of 0.25. Sequences of the differential ASVs as well as the absolute total reads per group are available in Table S3. The microbial taxonomic features (i.e., families or genera) most likely to explain differences between healthy subjects and patients suffering from UC were also determined by LEfSe (linear discriminant analysis effect size), using the packages “microbiomeMarker” and “yingtools2”.

## 3. Results

### 3.1. Clinical Characteristics of the Participants

Initially, we were able to recruit 20 UC patients and 11 healthy volunteers who agreed to participate in the study. After applying the strict inclusion and exclusion criteria, 10 UC patients dropped out of the study for various reasons (worsening of the disease or its symptoms, other diseases, antibiotics, or lack of interest in the study). We finally enrolled 10 UC patients in the active phase of the disease, categorized as “mild or moderate” by the medical personnel of the Hospital Digestive Service—a fact that was supported by the high fecal calprotectin levels detected, as clinical indicators of their disease activity (Table S1). Moreover, none of the participants showed or manifested oral symptoms or oral diseases at the time of sampling. The data of all participants, including the clinical characteristics collected from medical records, are shown in Table S1. No significant differences existed between the groups in terms of age, gender, body mass index (BMI), and main clinical parameters. Values for fecal calprotectin and partial Mayo score are specifically reported for UC patients, as clinical indicators of their disease activity [23] (Table S1).

### 3.2. Changes in the Biodiversity Indices between UC Patients and Healthy Subjects

**Figure 1:**
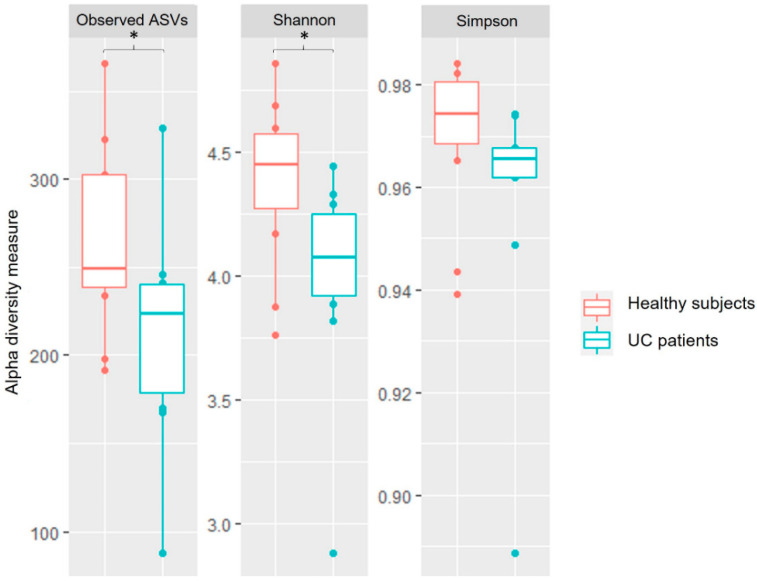
Boxplots representing the different alpha diversity indices (observed, Shannon, and Simpson) detected in the saliva samples of the UC patients and healthy subjects. Alpha indices that showed statistically differences (* *p*-value < 0.05) following the ANOVA test are marked with an asterisk.

**Figure 2:**
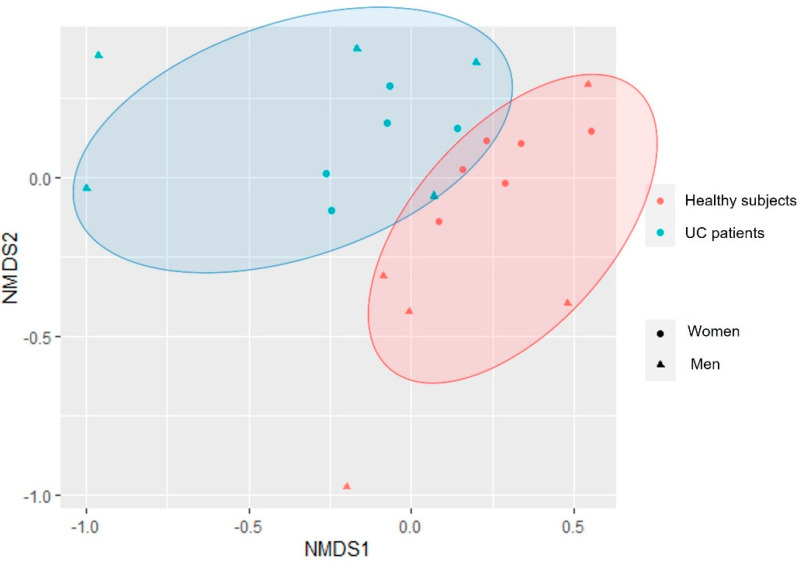
Beta diversity analysis following the NMDS Bray–Curtis method for the UC patients and healthy subjects.

Analysis of raw reads revealed a total of 3270 ASVs (amplicon sequence variants). Bacterial diversity analysis between groups showed a decrease in the alpha diversity indices in the UC patients, revealing statistically significant differences for the observed ASVs (*p*-value = 0.0454) and Shannon indices (*p*-value = 0.0314) (Figure 1), but not for the Simpson indices (*p*-value = 0.174). The analysis of the beta diversity showed two different groups (PERMANOVA, R2 = 0.06523, *p*-value = 0.08791): one for the healthy subjects, and another for the UC patients (Figure 2), with the female participants showing more similar microbial composition profiles, and the male participants a more scattered distribution, although this result did not show statistical significance (PERMANOVA, R2 = 0.06178, *p*-value = 0.14985).

### 3.3. Taxonomic Profiles of the UC Patients and Healthy Subjects

**Figure 3:**
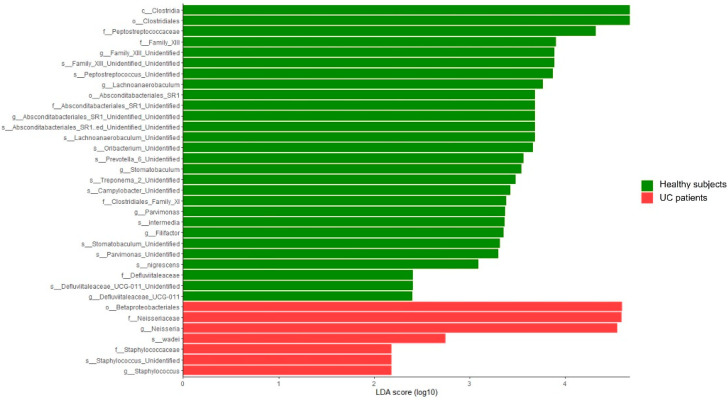
Linear discriminant analysis (LDA) scores (log10) for the significant taxonomic biomarkers obtained from the LEfSe analysis between the groups of healthy subjects and UC patients. Color code: green—taxa in higher proportions in the healthy group; red—taxa in higher proportions in UC participants.

Regarding the taxonomic profiles, results revealed differences in the relative abundance of some taxa between the group of healthy subjects and the UC patients (see Table S2 for all taxa, Table 1 for taxa with an average relative abundance > 0.5%, and Figure 3 for taxonomic biomarkers for each group calculated by LDA). At the phylum level, results revealed a tendency for higher relative proportions of Proteobacteria in the oral cavities of the UC patients (*p*-value = 0.072). In relation to the family level, the results revealed statistically significant differences (*p*-value < 0.05) in the proportions of Family XIII and *Peptostreptococcaceae*, which showed lower values in their relative abundance in the UC patients, and in *Neisseriaceae*, whose values were higher in the UC group. These remarks were corroborated by LDA analysis (Figure 3). Furthermore, a trend (*p*-value < 0.1) was detected for lower levels of *Atopobiaceae*, *Lachnospiraceae* and *Ruminococcaceae* members in UC subjects.

At the genus level, results showed higher relative proportions of *Neisseria* genus members (*p*-value < 0.05) in the oral cavities of the UC subjects, which were also detected in higher proportions in this group by LDA approach (Figure 3). On the other hand, saliva samples of the UC group presented lower levels of *Filifactor*, *Parvimonas*, *Lachnoanaerobaculum*, and *Stomatobaculum* (*p*-value < 0.05), together with a trend of lower levels of *Atopobium*, *Peptostreptococcus*, and *Ruminococcaceae*-UCG_014 genera (*p*-value < 0.1) (Table 1 and Table S2). Additionally, although with a low relative abundance (mean 0.005%), our results highlighted the exclusive presence of *Staphylococcus* members in the saliva samples of five of the UC patients (*p*-value = 0.011), with members of this genus not being detected in any of the healthy subjects (Table S2, Figure 3). LDA analysis revealed differences in the same taxa and bacterial groups, among other several unidentified species, supporting the statistical power of the results.

Finally, the analysis of the differences at the ASV level revealed 57 differential ASVs (*p*-value < 0.05), most of them only detected in the oral microbiomes of healthy donors (Table 2). In this regard, it is interesting to note the absence of different species of *Streptococcus*, *Oribacterium*, *Rothia*, *Prevotella*, and *Porphyromonas*, among others, in the saliva samples of the UC group. In particular, some species that were only detected in the saliva samples of the healthy donors’ group were ASVs assigned with high confidence/similarity to *Haemophilus parainfluenzae* and *Porphyromonas pasteri*, as well as *Veillonella atypica* and different species of the *Rothia* genus (e.g., *Rothia dentocariosa* and *Rothia mucilaginosa*)—many of them well-known members of the healthy oral core microbiome [24]. Nevertheless, on the other hand, four ASVs were only detected in the oral cavities of the UC group, highlighting ASV4, ASV14, ASV39, and ASV219. BLAST analysis of these ASVs showed that ASV4 and ASV14 had the highest similarity with *Veillonella parvula* (99.78% and 100% identity, respectively), ASV39 with *Fusobacterium nucleatum* (100% identity), and ASV219 with *Prevotella* sp. (100% identity).

## 4. Discussion

Currently, there are several studies describing the gut microbiome changes and a dysbiotic state in UC patients [4,25]. However, UC can be related to other extraintestinal symptoms, such as oral lesions or oral diseases, which can occur together with intestinal symptoms or even before they occur [5,8]. Various studies have reported oral manifestations in IBD patients [5,6,7,8,26,27,28,29,30], whose findings have led to the proposal of a “gum–gut” axis, revealing the importance of the balance in the oral microbial communities not only for oral health, but also for overall health status [27]. Moreover, it has been suggested that UC-related oral manifestations could be due to dysbiosis in the oral microbiome associated with this pathology. To the best of our knowledge, this is the first study characterizing the oral microbiota present in adult UC patients in terms of both composition and diversity. Additionally, and bearing in mind the intrinsic variability associated with oral microbiota, this study considered a quite homogeneous patient cohort, all with an active phase of UC, categorized as mild or moderate.

As expected, we found an important interindividual variability between the salivary microbial profiles of the participants of the study. However, our results indicated a decrease in alpha diversity indices in UC patients, in agreement with the observations of Zhang et al. during the active phase of CD, and the results of Docktor et al. and Elmaghrawy et al. in pediatric cohorts [2,13,15]. In the same line, Lucas López et al. described a decrease in alpha diversity indices in the gut microbiome of UC patients [3], so it can be hypothesized that UC could be associated with a loss of both gut and oral bacterial diversity. The analysis of the beta diversity showed two clearly differentiated groups, revealing that the taxonomic composition of the oral microbiomes of UC patients and healthy subjects is different. Potential sex differences in the oral microbial profiles, consistent with the pattern previously described in the gut microbiome of IBD subjects [31]—including some studies in murine models [32,33]—need to be tested with larger sample sizes.

At the phylum level, we observed a trend towards a higher relative abundance of Proteobacteria in the oral cavities of the UC patients, as previously reported in the gut microbiome of IBD patients [18,34,35]. At the family level, also consistent with our results, depletion in the levels of *Peptostreptococcaceae* and *Lachnospiraceae* has been reported in the oral cavities of UC or CD patients [13,18] and, in the case of *Lachnospiraceae* and *Ruminococcaceae* bacteria, also in the gut microbiome [36,37]. However, contrary to what has been described in previous studies [13,18,38], our results showed significantly higher values for *Neisseriaceae* family members and *Neisseria*—dominant commensal members in the healthy oral cavity [24,39], but which can also act as opportunistic pathogens [40,41]. Conversely, we detected lower proportions of *Atopobium*, *Lachnoanaerobaculum*, *Peptostreptococcus*, and *Stomatobaculum* members in the saliva samples of the UC patients. The majority of these members form part of the oral core microbiome [39,42], and some species—for example, *Stomatobaculum longum* and *Lachnoanaerobaculum saburreum*—have been related to oral health conditions [43,44,45]. Moreover, the depletion in *Peptostreptococcus* members as well as other oral commensals in the saliva samples of UC patients was previously reported by Xun et al. in the oral cavities of IBD patients [18], while lower proportions of *Peptostreptococcus* members were also reported in the gut microbiome of UC patients [46]. The tryptophan metabolite indoleacrylic acid—produced by gut commensal *Peptostreptococcus* species—has shown the ability to promote intestinal epithelial barrier function and mitigate inflammatory responses in a murine model [47]. Thus, in line with this, a possible hypothesis could be that the depletion in *Peptostreptococcus* members, together with the lower proportions of other key members in the guts and oral cavities of the UC patients—such as *Lachnospiraceae* and *Ruminococcaceae*—could be related to lower anti-inflammatory activities, which could promote or contribute to the characteristic inflammatory profile of these patients.

Interestingly, our results highlighted the exclusive presence of *Staphylococcus* members in the saliva samples in 5 of the 10 UC patients, but not in any of the healthy controls. *Staphylococcus* members are not part of the normal oral microbiota, but they have been found to occur in immunocompromised patients who can suffer from oral inflammatory conditions (e.g., gingivitis caused by *Staphylococcus epidermidis*) [48]. Although further evidence is needed due to the small sample size of this study, which could limit these results, the presence of members of the *Staphylococcus* genus in the oral cavities of UC patients could be proposed as an oral biomarker of the disease in a non-invasive sample.

Analysis of the differences at the ASV level between healthy donors and UC patients revealed the absence of different species/phylotypes belonging to key members of the oral core microbiome, such as members of *Prevotella* (i.e., *Prevotella salivae*, *Prevotella histicola*, and *Prevotella melaninogenica*), *Porphyromonas* (i.e., *Porphyromonas pasteri*), and *Rothia* (i.e., *Rothia dentocariosa*), in the saliva samples of UC patients—a finding that has been also reported in IBD patients [13,18]. Moreover, some species that were only detected in the saliva samples of the healthy donors’ group—such as *H. parainfluenzae*, *P. pasteri*, *V. atypica* *R. dentocariosa*, and *R. mucilaginosa*—have been correlated in other works with a healthy oral state [24,49,50,51]. On the other hand, four ASVs were only detected in the saliva samples of the UC patients, corresponding to *V. parvula*, *F. nucleatum*, and *Prevotella* sp. Members belonging to *Veillonella* are among the most abundant members of the oral microbiome [52], and their co-aggregation properties play a crucial role affecting the colonization site and the ecology of oral microbial biofilms [53]. Although *V. parvula* plays an essential role as a commensal in the oral microbiome, and is considered to be non-pathogenic, rarely causing serious infections, it is present at very high levels in dental carious lesions, being predominant at all stages of caries progression and contributing to caries development [54]. In this sense, *Veillonella* species mainly utilize lactate as their sole carbon source, producing acetate and propionate, so it has been proposed that their levels may serve as a sensitive biological indicator and early warning sign of acid production [55]. Taking this into account, a possible explanation for the higher levels of *V. parvula* in the UC patients could be that lactate is one of the most enriched byproducts of cellular metabolism in inflamed tissues, and its accumulation leads to the exacerbation of the inflammatory response [56]. The chronic inflammatory state present in UC could promote lactate production in the oral cavity, which, together with the higher levels of other acid-producing bacteria, could provide the conditions for the improved growth of lactate-utilizing bacteria such as *V. parvula* and *Neisseria* [57].

Regarding *F. nucleatum*, it is a common member of the oral core microbiome; however, because of its proinflammatory potential, it has been associated with various periodontal diseases, with its increase also related to the severity of disease and the progression of inflammation [58]. Furthermore, *F. nucleatum* has been detected as being significantly enriched in the gut in IBD and UC subjects [4,59,60], being related even to the promotion of colitis, its aggravation, and the impediment of the remission [61,62,63], as well as having been found to cause opportunistic infections, and to be associated with colorectal cancer and IBD [64].

Some of these differential oral species have been reported in higher proportions in the gut microbiome of UC patients, highlighting the previously mentioned *F. nucleatum* and *V. parvula*, which have been reported in increased levels in IBD patients [60,65]. Considering that in our study these bacterial species/phylotypes were only observed in the saliva samples of the UC patients, together with the exclusive detection of *Staphylococcus* members in this group, our results suggest that the presence or the increase in the proportions of these bacterial members could be used as possible oral biomarkers associated with UC. However, we are aware that the small sample size could limit the detection of these species in the control group, so these trends need to be corroborated in studies with larger numbers of participants.

By comparing our data to gut microbiome profiles previously described in UC patients, we observed that several of the trends observed in some oral microorganisms in our study were consistent with changes detected in the guts of the UC subjects, highlighting the slight increase in *Streptococcus* members in the oral cavity, as also reported in the gut microbiome with UC [66], as well as the previously mentioned higher proportions of *Gemella* and *Haemophilus* members [67], and the depletion of *Lachnospiraceae*, *Ruminococcaceae*, and *Prevotella* in UC [4,18,68,69]. Furthermore, some of the specific species detected in the oral microbiome of the UC patients’ group of our study were reported in higher levels in the gut microbiome of UC patients. Thus, a plausible explanation for our results is that the changes in the bacterial communities causing or caused by UC could be similar in the oral cavity and the gut, revealing a possible association between the microbial communities of the two environments and the risk of the disease. In general, oral bacteria are poor colonizers of the healthy intestine, although it is known that transmission and colonization of the intestine by oral microorganisms is common and extensive among healthy individuals [70]. Considering that we ingest about 1 L of saliva per day—containing an enormous amount of oral bacteria—it is possible that some members of the oral microbiome can tolerate the harsh pH of the stomach or can translocate to the gut, facilitated by gum inflammation, and then colonize and proliferate in the gastrointestinal tract, promoting gut dysbiosis. This is the case of *P. gingivalis*—which can resist acid pH, and can reach the gut by descending from the oral cavity, promoting a modulation of the gut microbiome in animals and humans, which is thought to have a role in orodigestive cancers [71]—and the colon-cancer-associated bacterium *F. nucleatum*, which reaches the colon from the oral cavity via the circulatory system [72,73]. Atarashi et al. revealed increased amounts of different typically oral bacteria in the fecal microbiota of UC patients, highlighting species belonging to the genera *Fusobacterium*, *Veillonella*, *Streptococcus*, *Neisseria*, *Prevotella*, and *Gemella* [74]. Some members belonging to these genera were observed in slightly higher proportions in the saliva samples of the UC participants of our study, so our results could suggest—supporting the previously hypothesized [74,75,76]—that some potential pathogenic members of an imbalanced oral microbiota could reach and colonize the gut and, consequently, promote a change in the gut microbial communities, leading to an aberrant activation of the immune system, and promoting the chronic gut inflammatory state associated with UC. On the other hand, our results reflect the possible changes in the saliva microbiome associated with UC, from an overall point of view. However, more frequent UC oral manifestations normally occur at specific mucosal sites; therefore, the analysis of possible changes in the microbiome of specific mucosal surfaces could provide more detailed information on the existence of a relationship between oral bacteria, UC oral manifestations, and UC itself.

## 5. Conclusions

In conclusion, our study suggests the presence of oral dysbiosis associated with UC, and supports the hypothesis that UC could be associated not only with gut dysbiosis, as previously observed, but also with an imbalance in the oral microbial communities, with the proportions of some commensals and opportunistic-pathogen-related groups altered in the oral cavity, and even with the possible presence of specific bacterial species/phylotypes that could be used, after corroboration in future studies with larger sample sizes, as oral biomarkers of UC. Taken together, these results provide a glimpse into the distinctive oral microbiome in ulcerative colitis, and allow for a deeper understanding of the oral health trends therein. Future large-scale studies are needed to corroborate the trends observed and provide insight into the possible role of the oral microbiome in the risk of ulcerative colitis.

## Figures and Tables

**Table 1:** Comparison of the oral taxonomic profiles between UC patients and healthy subjects. Only taxa with an average relative abundance > 0.5% are represented.

Taxonomy	Mean ± SD Healthy Subjects	Mean ± SD UC Patients	*p*-Value
Phylum			
Bacteroidetes	31.46 ± 10.18	33.88 ± 5.46	0.704
Firmicutes	41.90 ± 5.56	44.89 ± 6.88	0.511
Proteobacteria	11.49 ± 7.47	6.24 ± 4.35	0.072 ^#^
Patescibacteria	2.30 ± 1.69	3.75 ± 2.55	0.314
Fusobacteria	6.56 ± 3.68	5.29 ± 3.26	0.468
Actinobacteria	5.64 ± 3.57	4.77 ± 2.20	0.704
Epsilonbacteraeota	0.50 ± 0.33	0.69 ± 0.35	0.426
Family			
*Actinomycetaceae*	1.41 ± 0.71	1.3 ± 1.22	0.349
*Atopobiaceae*	1.08 ± 0.56	0.67 ± 0.66	0.084 ^#^
*Campylobacteraceae*	0.69 ± 0.35	0.50 ± 0.33	0.426
*Carnobacteriaceae*	1.27 ± 0.69	1.22 ± 0.55	0.972
*Erysipelotrichaceae*	0.54 ± 0.23	0.50 ± 0.53	0.217
*Family_XI*	1.06 ± 0.42	1.90 ± 1.30	0.132
*Family_XIII*	1.16 ± 0.89	0.35 ± 0.31	0.024 *
*Flavobacteriaceae*	0.68 ± 0.45	1.35 ± 1.31	0.511
*Fusobacteriaceae*	2.48 ± 1.47	2.96 ± 2.49	0.972
*Lachnospiraceae*	3.38 ± 1.45	2.00 ± 1.33	0.061 ^#^
*Leptotrichiaceae*	2.81 ± 2.18	3.60 ± 2.58	0.511
*Micrococcaceae*	2.20 ± 1.65	3.30 ± 2.14	0.251
*Neisseriaceae*	2.19 ± 1.58	6.29 ± 5.14	0.019 *
*Pasteurellaceae*	3.90 ± 2.83	5.00 ± 2.94	0.349
*Peptostreptococcaceae*	2.54 ± 4.02	0.38 ± 0.46	0.044 *
*Porphyromonadaceae*	5.92 ± 5.15	2.93 ± 2.83	0.223
*Prevotellaceae*	26.75 ± 8.24	26.76 ± 10.43	1
*Ruminococcaceae*	0.59 ± 0.42	0.24 ± 0.22	0.066 ^#^
*Saccharimonadaceae*	2.84 ± 1.98	2.02 ± 1.77	0.426
*Streptococcaceae*	11.77 ± 4.98	12.29 ± 6.20	0.863
*Veillonellaceae*	21.86 ± 7.26	22.24 ± 7.52	0.917
Genus			
*Actinomyces*	1.40 ± 0.72	1.28 ± 1.20	0.387
*Alloprevotella*	3.00 ± 1.53	3.15 ± 2.10	0.918
*Atopobium*	1.07 ± 0.56	0.67 ± 0.66	0.084 ^#^
*Campylobacter*	0.69 ± 0.35	0.50 ± 0.33	0.426
*Capnocytophaga*	0.68 ± 0.45	1.35 ± 1.31	0.511
*Fusobacterium*	2.48 ± 1.47	2.96 ± 2.49	0.972
*Gemella*	1.06 ± 0.42	1.90 ± 1.30	0.132
*Granulicatella*	1.27 ± 0.69	1.22 ± 0.55	0.972
*Haemophilus*	3.73 ± 2.78	4.66 ± 2.74	0.386
*Lachnoanaerobaculum*	0.95 ± 0.60	0.33 ± 0.19	0.018 *
*Leptotrichia*	2.78 ± 2.15	3.60 ± 2.58	0.511
*Megasphaera*	1.65 ± 1.57	1.06 ± 1.02	0.573
*Neisseria*	2.01 ± 1.54	5.66 ± 5.15	0.034 *
*Oribacterium*	1.25 ± 0.73	0.96 ± 0.69	0.511
*Peptostreptococcus*	2.19 ± 3.85	0.34 ± 0.43	0.084 ^#^
*Porphyromonas*	5.92 ± 5.15	2.93 ± 2.83	0.223
*Prevotella*	4.18 ± 2.39	3.48 ± 2.27	0.756
*Prevotella*_6	1.20 ± 0.75	0.93 ± 0.86	0.314
*Prevotella*_7	18.1 ± 8.54	18.83 ± 9.34	0.917
*Rothia*	2.20 ± 1.65	3.30 ± 2.14	0.251
*Ruminococcaceae*_UCG-014	0.58 ± 0.42	0.24 ± 0.22	0.066 ^#^
*Selenomonas_3*	0.88 ± 0.84	0.67 ± 0.71	0.459
*Solobacterium*	0.52 ± 0.24	0.50 ± 0.53	0.245
*Stomatobaculum*	0.59 ± 0.37	0.24 ± 0.18	0.035 *
*Streptococcus*	11.77 ± 4.98	12.29 ± 6.2	0.863
*Veillonella*	18.89 ± 7.41	20.18 ± 7.8	0.704

* *p*-value < 0.05, ^#^ *p*-value < 0.1.

**Table 2:** Analysis of the differential relative abundance (%) at the ASV level: Only ASVs with statistically significant differences (*p*-value < 0.05) based on the Mann–Whitney U test for non-related samples are shown. The ASVs that were detected only in the group of UC patients are marked in red, and the ASVs that were detected exclusively in the group of healthy donors are marked in green.

ASV Code	Species	Mean ± SD Healthy Subjects	Mean ± SD UC Patients	*p*-Value
ASV4	*Veillonella parvula*	ND	0.136 ± 0.296	-
ASV14	*Veillonella parvula*	ND	0.152 ± 0.316	-
ASV39	*Fusobacterium nucleatum*	ND	0.068 ± 0.118	-
ASV219	*Prevotella* NA	ND	0.01 ± 0.019	-
ASV754	*Haemophilus parainfluenzae*	0.771 ± 0.896	ND	-
ASV756	*Saccharimonadaceae* family NA	0.742 ± 0.591	ND	-
ASV758	*Rothia mucilaginosa*	0.567 ± 0.626	0.162 ± 0.326	3.65 × 10^−2^
ASV762	*Veillonella rogosae*	0.704 ± 1.004	ND	-
ASV763	*Porphyromonas* NA	0.530 ± 0.823	ND	-
ASV765	*Veillonella atypica*	0.690 ± 0.469	ND	-
ASV773	*Prevotella histicola*	0.519 ± 0.573	ND	-
ASV778	*Veillonella rogosae*	0.356 ± 0.440	ND	-
ASV781	*Prevotella melaninogenica*	0.390 ± 0.978	ND	-
ASV787	*Rothia mucilaginosa*	0.246 ± 0.434	1.419 ± 1.304	2.72 × 10^−2^
ASV792	*Porphyromonas* NA	0.270 ± 0.271	ND	-
ASV795	*Peptostreptococcus stomatis*	0.793 ± 1.565	ND	-
ASV797	*Streptococcus salivarius*	0.453 ± 0.736	ND	-
ASV809	*Prevotella salivae*	0.245 ± 0.225	ND	-
ASV814	*Ruminococcaceae*_UCG-014 NA	0.247 ± 0.224	0.071 ± 0.103	3.43 × 10^−2^
ASV816	*Prevotella jejuni*	0.269 ± 0.381	ND	-
ASV821	*Oribacterium sinus*	0.199 ± 0.225	ND	-
ASV834	*Eubacterium sulci*	0.264 ± 0.270	ND	-
ASV842	*Prevotella nanceiensis*	0.139 ± 0.323	ND	-
ASV852	*Gemella sanguinis*	0.121 ± 0.101	ND	-
ASV855	*Porphyromonas pasteri*	0.170 ± 0.290	ND	-
ASV857	*Alloprevotella* NA	0.302 ± 0.687	ND	-
ASV859	*Prevotella nanceiensis*	0.121 ± 0.216	ND	-
ASV861	*Bergeyella* NA	0.093 ± 0.042	0.18 ± 0.115	2.42 × 10^−2^
ASV865	*Absconditabacteriales*_SR1 order NA	0.162 ± 0.223	ND	-
ASV875	*Leptotrichia* NA	0.191 ± 0.378	ND	-
ASV878	*Campylobacter concisus*	0.105 ± 0.071	ND	-
ASV883	*Lachnoanaerobaculum gingivalis*	0.125 ± 0.144	ND	-
ASV893	*Oribacterium parvum*	0.115 ± 0.135	ND	-
ASV894	*Granulicatella elegans*	0.043 ± 0.093	ND	-
ASV896	*Parvimonas micra*	0.059 ± 0.089	ND	-
ASV901	*Rothia dentocariosa*	0.082 ± 0.123	ND	-
ASV905	*Ruminococcaceae*_UCG-014 NA	0.089 ± 0.096	ND	-
ASV906	*Leptotrichia* NA	0.099 ± 0.142	ND	-
ASV920	*Streptococcus sanguinis*	0.068 ± 0.060	ND	-
ASV964	*Prevotella melaninogenica*	0.098 ± 0.204	ND	-
ASV967	*Stomatobaculum longum*	0.097 ± 0.177	ND	-
ASV979	*Catonella morbi*	0.043 ± 0.064	ND	-
ASV1001	*Oribacterium asaccharolyticum*	0.028 ± 0.040	ND	-
ASV1010	*Lachnospiraceae* family NA	0.064 ± 0.084	0.013 ± 0.029	3.02 × 10^−2^
ASV1015	*Treponema*_2 NA	0.032 ± 0.045	ND	-
ASV1050	*Eubacterium yurii*	0.058 ± 0.137	ND	-
ASV1101	*Capnocytophaga* NA	0.026 ± 0.040	ND	-
ASV1153	*Campylobacter gracilis*	0.043 ± 0.089	ND	-
ASV1158	*Capnocytophaga sputigena*	0.039 ± 0.070	ND	-
ASV1161	*Selenomonas*_3 NA	0.027 ± 0.038	ND	-
ASV1329	*Streptococcus sanguinis*	0.032 ± 0.071	ND	-
ASV1363	*Prevotella* NA	0.003 ± 0.007	ND	-
ASV1405	*Treponema refringens*	0.015 ± 0.025	ND	-
ASV1414	*Streptococcus gordonii*	0.012 ± 0.023	ND	-
ASV1473	*Butyrivibrio*_2 NA	0.015 ± 0.035	ND	-
ASV1569	*Candidatus_Saccharimonas* NA	0.013 ± 0.021	ND	-
ASV1600	*Fretibacterium* NA	0.024 ± 0.052	ND	-

ND = not detected.

## Data Availability

The FASTQ files for the samples used for the analyses in this study were uploaded to the Sequence Read Archive (SRA) with the BioProject accession number PRJNA749643, and can be found here: http://www.ncbi.nlm.nih.gov/bioproject/749643, last accessed on 28 July 2021.

## Supplementary Materials

The following are available online at https://www.mdpi.com/article/10.3390/cimb44040103/s1, Figure S1: Plot showing error rates for each possible transition (A→C, A→G, …) (Points are the observed error rates for each consensus quality score). The black line shows the estimated error rates after convergence of the machine-learning algorithm. The red line shows the error rates expected under the nominal definition of the Q-score. Here the estimated error rates (black line) are a good fit to the observed rates (points), and the error rates drop with increased quality as expected, Figure S2: Rarefaction curves per sample that show the sequencing depth of the study, Table S1: Clinical information (mean ± SD or range) of the participants in this study, Table S2: Comparison of the oral taxonomic profiles between UC participants of the study and a group of healthy donors, Table S3: Sequence of the differential ASVs detected between UC patients and healthy volunteers. Absolute number of reads detected per sample (number codes) in each group is also shown, Table S4: Description of the sequencing run and processing.
