# Supplementary material for: Ulcerative Colitis Seems to Imply Oral Microbiome Dysbiosis
Source: Curr Issues Mol Biol. 2022 Mar 30;44(4):1513–27. doi: 10.3390/cimb44040103 (PMC9164047; doi:10.3390/cimb44040103)
Supplement: Supplementary file 1 [file cimb-44-00103-s001.zip › cimb-1630639-supplementary.pdf]

Supplementary materials

Table S1. Clinical information (mean ± SD or range) of the participants in this study.

	**Healthy Subjects**	**Ulcerative Colitis Subjects**
**n (total)**	11	10
**n (females)**	6	5
**n (males)**	5	5
**Age (years)**	20 - 44	18 - 42
**BMI (18.5-24.9 Kg/ m²)**	23.66 ± 4.17	26.94 ± 4.70
**Glucose (70-110mg/gL)**	77.63 ± 5.86	109.00 ± 52.05
**Cholesterol (<200 mg/gL)**	180.90 ± 23.92	202.50 ± 19.82
**Triglycerides (<150 mg/gL)**	76.54 ± 21.40	118.75 ± 56.75
**HDL (>40 mg/gL)**	61.54 ± 12.63	52.50 ± 3.50
**LDL (<130 mg/gL)**	104.18 ± 24.34	127.00 ± 33.00
**Fecal calprotectin (<50 μg/g)**	-	1827.94 ± 2159.58
**Partial Mayo score**	-	4.22 ± 1.61

BMI, Body Mass Index; HDL, High Density Lipoprotein; LDL, Low Density Lipoprotein. Partial Mayo score is calculated from "Stool frequency", "Rectal Bleeding" and Physician's global assessment". The range values considered normal for each value are shown in brackets.

Table S2. Comparison of the oral taxonomic profiles between UC participants of the study and a group of healthy donors.

**Taxonomy**	**Mean ± SD Healthy Volunteers**	**Mean ± SD UC Patients**	***p-values***
Phylum			
Actinobacteria	4.77 ± 2.20	5.64 ± 3.57	0.704
Bacteroidetes	33.88 ± 5.46	31.46 ± 10.18	0.704
Bacteroidetes	33.88 ± 5.46	31.46 ± 10.18	0.704
Epsilonbacteraeota	0.69 ± 0.35	0.50 ± 0.33	0.426

Firmicutes	44.89 ± 6.88	41.9 ± 5.56	0.511
Fusobacteria	5.29 ± 3.26	6.56 ± 3.68	0.468
Patescibacteria	3.75 ± 2.55	2.30 ± 1.69	0.314
**Proteobacteria**	**6.24 ± 4.35**	**11.49 ± 7.47**	**0.072#**
**Spirochaetes**	**0.39 ± 0.80**	**0.09 ± 0.12**	**0.097#**
Synergistetes	0.04 ± 0.08	0.01 ± 0.03	0.294
Tenericutes	0.05 ± 0.05	0.03 ± 0.05	0.287
Family			
Actinomycetaceae	1.41 ± 0.71	1.30 ± 1.22	0.349
Aerococcaceae	0.05 ± 0.06	0.09 ± 0.15	0.802
Atopobiaceae	**1.08 ± 0.56**	**0.67 ± 0.66**	**0.084#**
Bacteroidaceae	0.03 ± 0.11	0.00 ± 0.00	1
Bifidobacteriaceae	0.03 ± 0.07	0.02 ± 0.04	0.310
Burkholderiaceae	0.08 ± 0.08	0.14 ± 0.12	0.481
Campylobacteraceae	0.69 ± 0.35	0.50 ± 0.33	0.426
Cardiobacteriaceae	0.02 ± 0.02	0.05 ± 0.05	0.319
Carnobacteriaceae	1.27 ± 0.69	1.22 ± 0.55	0.972
Corynebacteriaceae	0.04 ± 0.03	0.33 ± 0.55	0.129
Defluviitaleaceae	**0.02 ± 0.03**	**0.00 ± 0.00**	**0.014***
Erysipelotrichaceae	0.54 ± 0.23	0.50 ± 0.53	0.217
Family_XI	1.06 ± 0.42	1.90 ± 1.30	0.132
**Family_XI**	**0.35 ± 0.24**	**0.23 ± 0.50**	**0.022***
**Family_XIII**	**1.16 ± 0.89**	**0.35 ± 0.31**	**0.024***
Flavobacteriaceae	0.68 ± 0.45	1.35 ± 1.31	0.511
Fusobacteriaceae	2.48 ± 1.47	2.96 ± 2.49	0.972
Lachnospiraceae	**3.38 ± 1.45**	**2.00 ± 1.33**	**0.061#**
Lactobacillaceae	0.12 ± 0.33	0.37 ± 0.74	0.342

Lentimicrobiaceae	0.02 ± 0.02	0.02 ± 0.05	0.172
Leptotrichiaceae	2.81 ± 2.18	3.60 ± 2.58	0.511
Micrococcaceae	2.20 ± 1.65	3.30 ± 2.14	0.251
Moraxellaceae	0.04 ± 0.10	0.01 ± 0.02	0.508
Mycoplasmataceae	0.02 ± 0.02	0.03 ± 0.05	0.732
Neisseriaceae	**2.19 ± 1.58**	**6.29 ± 5.14**	**0.019***
Paludibacteraceae	0.07 ± 0.12	0.04 ± 0.06	0.643
Pasteurellaceae	3.90 ± 2.83	5.00 ± 2.94	0.349
Peptococcaceae	**0.16 ± 0.15**	**0.08 ± 0.16**	**0.064#**
Peptostreptococcaceae	**2.54 ± 4.02**	**0.38 ± 0.46**	**0.044***
Porphyromonadaceae	5.92 ± 5.15	2.93 ± 2.83	0.223
Prevotellaceae	26.75 ± 8.24	26.76 ± 10.43	1
Propionibacteriaceae	0.00 ± 0.00	0.02 ± 0.04	0.147
Rikenellaceae	**0.05 ± 0.12**	**0.00 ± 0.00**	**0.057#**
Ruminococcaceae	**0.59 ± 0.42**	**0.24 ± 0.22**	**0.066#**
Saccharimonadaceae	2.84 ± 1.98	2.02 ± 1.77	0.426
Spirochaetaceae	**0.39 ± 0.80**	**0.09 ± 0.12**	**0.096#**
Streptococcaceae	11.77 ± 4.98	12.29 ± 6.20	0.863
Synergistaceae	0.04 ± 0.08	0.01 ± 0.03	0.294
Tannerellaceae	0.16 ± 0.11	0.13 ± 0.10	0.698
Veillonellaceae	21.86 ± 7.26	22.24 ± 7.52	0.917
Weeksellaceae	0.16 ± 0.16	0.23 ± 0.15	0.173
Genus			
Abiotrophia	0.050 ± 0.055	0.090 ± 0.150	0.802
Acholeplasma	0.006 ± 0.016	0.000 ± 0.000	0.189
Actinobacillus	0.007 ± 0.021	0.029 ± 0.052	0.281
Actinomyces	1.397 ± 0.721	1.279 ± 1.202	0.387

Aggregatibacter	0.085 ± 0.084	0.206 ± 0.294	0.971
Alloprevotella	3.003 ± 1.527	3.148 ± 2.102	0.918
Alloscardovia	0.001 ± 0.002	0.002 ± 0.007	0.729
Alysiella	0.008 ± 0.014	0.006 ± 0.012	0.814
Anaeroglobus	0.025 ± 0.029	0.014 ± 0.020	0.469
Atopobium	**1.068 ± 0.563**	**0.673 ± 0.656**	**0.084**[#]
Bacteroides	0.035 ± 0.109	0.000 ± 0.000	1
Bergeriella	0.001 ± 0.002	0.000 ± 0.000	0.391
Bergeyella	0.163 ± 0.159	0.225 ± 0.152	0.173
Bifidobacterium	0.016 ± 0.027	0.006 ± 0.014	0.257
Bulleidia	**0.009 ± 0.017**	**0.000 ± 0.000**	**0.093**[#]
*Butyrivibrio*_2	0.137 ± 0.107	0.183 ± 0.320	0.454
Campylobacter	0.686 ± 0.351	0.501 ± 0.327	0.426
Candidatus_Saccharimonas	0.321 ± 0.216	0.240 ± 0.236	0.503
Capnocytophaga	0.681 ± 0.449	1.351 ± 1.311	0.511
Cardiobacterium	0.020 ± 0.016	0.049 ± 0.053	0.318
Catonella	0.208 ± 0.138	0.190 ± 0.153	0.860
Citrobacter	0.001 ± 0.002	0.000 ± 0.000	0.391
Comamonas	0.001 ± 0.004	0.000 ± 0.000	0.391
Corynebacterium	0.040 ± 0.027	0.332 ± 0.547	0.128
Cryptobacterium	0.001 ± 0.002	0.002 ± 0.005	0.729
***Defluviitaleaceae*_UCG-011**	**0.022 ± 0.031**	**0.001 ± 0.004**	**0.014***
Desulfobulbus	0.001 ± 0.002	0.000 ± 0.000	0.391
Dialister	0.122 ± 0.128	0.108 ± 0.110	0.503
Eggerthia	0.004 ± 0.012	0.000 ± 0.000	0.391
Eikenella	0.020 ± 0.023	0.032 ± 0.034	0.424
*Erysipelotrichaceae*_UCG-006	0.001 ± 0.003	0.001 ± 0.003	0.945

Ezakiella	**0.007 ± 0.014**	**0.000 ± 0.000**	**0.093#**
Family_XIII_UCG-001	0.032 ± 0.039	0.008 ± 0.014	0.138
Filifactor	**0.218 ± 0.428**	**0.014 ± 0.033**	**0.014****
Fretibacterium	0.034 ± 0.071	0.015 ± 0.030	0.294
Fusobacterium	2.475 ± 1.472	2.962 ± 2.493	0.972
Gemella	1.061 ± 0.424	1.903 ± 1.302	0.132
Granulicatella	1.266 ± 0.689	1.218 ± 0.554	0.972
Haemophilus	3.728 ± 2.775	4.658 ± 2.739	0.386
Howardella	0.001 ± 0.004	0.001 ± 0.002	1
Johnsonella	0.138 ± 0.175	0.059 ± 0.093	0.441
Kingella	0.050 ± 0.042	0.488 ± 1.184	0.417
Lachnoanaerobaculum	**0.947 ± 0.599**	**0.333 ± 0.187**	**0.018***
*Lachnospiraceae*_NK3A20_group	0.002 ± 0.006	0.000 ± 0.000	0.391
Lactobacillus	0.118 ± 0.325	0.373 ± 0.739	0.342
Lautropia	0.071 ± 0.080	0.106 ± 0.118	0.831
Leptotrichia	2.784 ± 2.154	3.595 ± 2.582	0.511
Megasphaera	1.653 ± 1.565	1.065 ± 1.022	0.573
Mobiluncus	0.015 ± 0.023	0.008 ± 0.016	0.402
Mogibacterium	0.010 ± 0.014	0.002 ± 0.005	0.136
Moraxella	0.037 ± 0.103	0.007 ± 0.019	0.741
Moryella	0.007 ± 0.023	0.000 ± 0.000	0.391
Mycoplasma	0.018 ± 0.022	0.026 ± 0.047	0.732
Neisseria	**2.013 ± 1.544**	**5.664 ± 5.148**	**0.034***
Olsenella	0.012 ± 0.039	0.000 ± 0.000	0.391
Oribacterium	1.250 ± 0.731	0.961 ± 0.685	0.511
Parascardovia	0.002 ± 0.005	0.000 ± 0.000	0.391
Parvimonas	**0.338 ± 0.238**	**0.233 ± 0.498**	**0.022***

Peptoanaerobacter	0.003 ± 0.008	0.004 ± 0.006	0.895
Peptococcus	**0.162 ± 0.149**	**0.075 ± 0.165**	**0.064#**
Peptoniphilus	0.001 ± 0.002	0.000 ± 0.000	0.391
Peptostreptococcus	**2.189 ± 3.849**	**0.341 ± 0.434**	**0.084#**
Phocaeicola	0.015 ± 0.045	0.000 ± 0.000	0.189
Porphyromonas	5.921 ± 5.146	2.927 ± 2.826	0.223
Prevotella	4.179 ± 2.386	3.475 ± 2.266	0.756
*Prevotella*_2	0.240 ± 0.326	0.364 ± 0.360	0.672
*Prevotella*_6	1.196 ± 0.746	0.934 ± 0.862	0.314
*Prevotella*_7	18.104 ± 8.544	18.83 ± 9.337	0.917
Pseudomonas	0.000 ± 0.000	0.002 ± 0.003	0.147
Pseudopropionibacterium	0.000 ± 0.000	0.017 ± 0.043	0.147
Pseudoramibacter	0.005 ± 0.012	0.000 ± 0.000	0.189
Pyramidobacter	0.009 ± 0.029	0.000 ± 0.000	0.391
Ralstonia	0.008 ± 0.008	0.032 ± 0.063	0.633
***Rikenellaceae*_RC9_gut_group**	**0.047 ± 0.121**	**0.001 ± 0.002**	**0.057#**
Rothia	2.203 ± 1.655	3.296 ± 2.135	0.251
***Ruminococcaceae*_UCG-014**	**0.585 ± 0.423**	**0.239 ± 0.220**	**0.066#**
Scardovia	0.015 ± 0.042	0.009 ± 0.021	0.801
Selenomonas	0.193 ± 0.176	0.150 ± 0.152	0.479
*Selenomonas*_3	0.877 ± 0.844	0.674 ± 0.714	0.459
*Selenomonas*_4	0.028 ± 0.043	0.016 ± 0.020	0.797
Shuttleworthia	0.017 ± 0.025	0.008 ± 0.014	0.279
Simonsiella	0.003 ± 0.01	0.012 ± 0.027	0.524
Solobacterium	0.523 ± 0.241	0.500 ± 0.532	0.245
Sphaerochaeta	0.005 ± 0.016	0.000 ± 0.000	0.391
Staphylococcus	**0.000 ± 0.000**	**0.005 ± 0.005**	**0.011***

Stomatobaculum	**0.592 ± 0.374**	**0.242 ± 0.177**	**0.035***
Streptobacillus	0.008 ± 0.014	0.002 ± 0.006	0.281
Streptococcus	11.77 ± 4.984	12.291 ± 6.196	0.863
Tannerella	0.160 ± 0.115	0.132 ± 0.098	0.698
Treponema_2	**0.389 ± 0.781**	**0.094 ± 0.116**	**0.096[#]**
Veillonella	18.892 ± 7.413	20.180 ± 7.801	0.704

Table S3. Sequence of the differential ASVs detected between UC patients and healthy volunteers. Absolute number of reads detected per sample (number codes) in each group is also shown.

		**Total number of reads detected per sample (codes) in UC group**										**Total number of reads detected per sample (codes) in Control group**										
**ASV Code Assigned**	**Sequence**	**116**	**118**	**120**	**122**	**124**	**126**	**128**	**130**	**132**	**134**	**56**	**58**	**60**	**65**	**67**	**69**	**73**	**75**	**77**	**82**	**85**
**ASV4**	GCAGCAGTGGGGAATCTTCCGCAATGGACGA<br>AAGTCTGACGGAGCAACGCCGCGTGAGTGAT<br>GACGGCCTTCGGGTTGTAAAGCTCTGTTAATC<br>GGGACGAAAGGCCTTCTTGCGAATAGTGAGA<br>AGGATTGACGGTACCGGAATAGAAAGCCACG<br>GCTAACTACGTGCCAGCAGCCGCGGTAATACG<br>TAGGTGGCAAGCGTTGTCCGGAATTATTGGGC<br>GTAAAGCGCGCGCAGGCGGATCAGTCAGTCT<br>GTCTTAAAAGTTCGGGGCTTAACCCCGTGATG<br>GGATGGAAACTGCTGATCTAGAGTATCGGAGA<br>GGAAAGTGGAATTCCTAGTGTAGCGGTGAAAT<br>GCGTAGATATTAGGAAGAACACCAGTGGCGA	0	16	85	468	65	0	0	0	0	64	0	0	0	0	0	0	0	0	0	0	0

	AGGCGACTTTCTGGACGAAAACTGACGCTGAGGCGCGAAAGCCAGGGGAGCGAACGGGATTAGATAC																					
**ASV14**	GCAGCAGTGGGGAATCTTCCGCAATGGACGAAAGTCTGACGGAGCAACGCCGCGTGAGTGATGACGGCCTTCGGGTTGTAAAGCTCTGTTAATCGGGACGAAAGGCCTTCTTGCGAACAGTTAGAAGGATTGACGGTACCGGAATAGAAAGCCACGGCTAACTACGTGCCAGCAGCCGCGGTAATACGTAGGTGGCAAGCGTTGTCCGGAATTATTGGGCGTAAAGCGCGCGCAGGCGGATCAGTTAGTCTGTCTTAAAAGTTCGGGGCTTAACCCCGTGATGGGATGGAAACTGCTGATCTAGAGTATCGGAGAGGAAAGTGGAATTCCTAGTGTAGCGGTGAAATGCGTAGATATTAGGAAGAACACCAGTGGCGAAGGCGACTTTCTGGACGAAAACTGACGCTGAGGCGCGAAAGCCAGGGGAGCGAACGGGATTAGATAC	0	495	89	106	0	20	0	0	0	0	0	0	0	0	0	0	0	0	0	0	0
**ASV39**	GCAGCAGTGGGGAATATTGGACAATGGACCAAGAGTCTGATCCAGCAATTCTGTGTGCACGATGAAGTTTTTCGGAATGTAAAGTGCTTTCAGTTGGGAAGAAAAAAATGACGGTACCAACAGAAGAAGTGACGGCTAAATACGTGCCAGCAGCCGCGGTAATACGTATGTCACAAGCGTTATCCGGATTTATTGGGCGTAAAGCGCGTCTAGGTGGTTATGTAAGTCTGATGTGAAAATGCAGGGCTCAACTCTGTATTGCGTTGGAAACTGTGTAACTAGAGT	0	38	0	116	199	4	0	0	0	0	0	0	0	0	0	0	0	0	0	0	0

	ACTGGAGAGGTAAGCGGAACTACAAGTGTAG AGGTGAAATTCGTAGATATTTGTAGGAATGCC GATGGGGAAGCCAGCTTACTGGACAGATACT GACGCTAAAGCGCGAAAGCGTGGGTAGCAAA CAGGATTAGATAC																					
**ASV219**	GCAGCAGTGAGGAATATTGGTCAATGGACGA AAGTCTGAACCAGCCAAGTAGCGTGCAGGAT GACGGCCCTCCGGGTTGTAAACTGCTTTTAGTT GGGAATAAAAAAAGGGACGTGTCCCTTATTGT ATGTACCTTCAGAAAAAGGACCGGCTAATTCC GTGCCAGCAGCCGCGGTAATACGGAAGGTCC AGGCGTTATCCGGATTTATTGGGTTTAAAGGG AGCGTAGGCGGATTGTTAAGTCAGCGGTTAAA GGGTGTGGCTCAACCATGCATTGCCGTTGAAA CTGGCGATCTTGAGTGCAGACAGGGATGCCGG AATTCGTGGTGTAGCGGTGAAATGCTTAGATA TCACGAAGAACTCCGATCGCGAAGGCAGGTG TCCGGGCTGCAACTGACGCTGAGGCTCGAAAG TGTGGGTATCAAACAGGATTAGATAC	28	0	0	0	0	0	13	3	0	9	0	0	0	0	0	0	0	0	0	0	0
**ASV754**	GCTGCAGTGGGGAATATTGCGCAATGGGGGC AACCCTGACGCAGCCATGCCGCGTGAATGAA GAAGGCCTTCGGGTTGTAAAGTTCTTTCGGTA GCGAGGAAGGCATTTAGTTTAATAGACTAGGT GATTGACGTTAACTACAGAAGAAGCACCGGCT AACTCCGTGCCAGCAGCCGCGGTAATACGGA GGGTGCGAGCGTTAATCGGAATAACTGGGCGT AAAGGGCACGCAGGCGGTGACTTAAGTGAGG	0	0	0	0	0	0	0	0	0	0	140	44	148	87	416	320	125	170	105	850	1764

	TGTGAAAGCCCCGGGCTTAACCTGGGAATTGC<br>ATTTCATACTGGGTCGCTAGAGTACTTTAGGG<br>AGGGGTAGAATTCCACGTGTAGCGGTGAAATG<br>CGTAGAGATGTGGAGGAATACCGAAGGCGAA<br>GGCAGCCCCTTGGGAATGTACTGACGCTCATG<br>TGCGAAAGCGTGGGGAGCAAACAGGATTAGA<br>TAC																					
**ASV756**	GCTGCAGTGAGGAATCTTCCACAATGGGCGAA<br>AGCCTGATGGAGCAACGCCGCGTGAAGGATG<br>AAGGCCTTCGGGTTGTAAACTTCTTTTATGAGT<br>GAAGAATATGACGGTAACTCATGAATAAGCA<br>CCGGCTAACTACGTGCCAGCAGCCGCGGTCAT<br>ACGTAGGGTGCAAGCATTATCCGGAGTGACTG<br>GGCGTAAAGAGTTGCGTAGGCGGTTTAATAAG<br>TGAATAGTGAAACCTGGTGGCTCAACCATACA<br>GACTATTATTCAAACTGTTAAACTCGAGAATG<br>GTAGAGGTAACTGGAATTTCTAGTGTAGGAGT<br>GAAATCCGTAGATATTAGAAGGAACACCGAT<br>GGCGTAGGCAGGTTACTGGGCCATTTCTGACG<br>CTAAGGCACGAAAGCGTGGGGAGCGAACCGG<br>ATTAGATAC	0	0	0	0	0	0	0	0	0	0	0	299	102	121	269	689	506	90	136	1081	964
**ASV758**	GCAGCAGTGGGGAATATTGCACAATGGGCGC<br>AAGCCTGATGCAGCGACGCCGCGTGAGGGAT<br>GACGGCCTTCGGGTTGTAAACCTCTGTTAGCA<br>GGGAAGAAGAGAGATTGACGGTACCTGCAGA<br>GAAAGCGCCGGCTAACTACGTGCCAGCAGCC<br>GCGGTAATACGTAGGGCGCGAGCGTTGTCCGG	0	398	0	0	0	0	434	0	0	0	44	0	651	0	296	71	0	66	904	502	459

	AATTATTGGGCGTAAAGAGCTTGTAGGCGGTTTGTCGCGTCTGCTGTGAAAGGCCGGGGCTTAACTCCGTGTATTGCAGTGGGTACGGGCAGACTAGAGTGCAGTAGGGGAGACTGGAATTCCTGGTGTAGCGGTGGAATGCGCAGATATCAGGAGGAACACCGATGGCGAAGGCAGGTCTCTGGGCTGTAACTGACGCTGAGAAGCGAAAGCATGGGGAGCGAACAGGATTAGATAC																					
**ASV762**	GCTGCAGTGGGGAATCTTCCGCAATGGACGAAAGTCTGACGGAGCAACGCCGCGTGAGTGATGACGGCCTTCGGGTTGTAAAGCTCTGTTAATCGGGACGAAAGGTCCTCTTGCGAATAGTTAGAGGAATTGACGGTACCGGAATAGAAAGCCACGGCTAACTACGTGCCAGCAGCCGCGGTAATACGTAGGTGGCAAGCGTTGTCCGGAATTATTGGGCGTAAAGCGCGCGCAGGCGGATCAGTCAGTCTGTCTTAAAAGTTCGGGGCTTAACCCCGTGATGGGATGGAAACTGCTGATCTAGAGTATCGGAGAGGAAAGTGGAATTCCTAGTGTAGCGGTGAAATGCGTAGATATTAGGAAGAACACCAGTGGCGAAGGCGACTTTCTGGACGAAAACTGACGCTGAGGCGCGAAAGCCAGGGGAGCGAACGGGATTAGATAC	0	0	0	0	0	0	0	0	0	0	0	0	0	595	879	77	77	71	0	688	1853
**ASV763**	GCTGCAGTGAGGAATATTGGTCAATGGGCGAGAGCCTGAACCAGCCAAGTCGCGTGAAGGATGACTGTCTTATGGATTGTAAACTTCTTTTATACGGGAATAACAAGAGCCACGTGTGGCTCCCTGC	0	0	0	0	0	0	0	0	0	0	0	23	0	0	1832	67	140	34	37	556	762

	ATGTACCGTATGAATAAGCATCGGCTAACTCCGTGCCAGCAGCCGCGGTAATACGGAGGATGCGAGCGTTATCCGGATTTATTGGGTTTAAAGGGTGCGTAGGCGGCCTGTTAAGTCAGCGGTGAAATCTAGGAGCTTAACTCCTAAATTGCCATTGATACTGGCGGGCTTGAGTGTAGATGAGGTAGGCGGAATGCGTGGTGTAGCGGTGGAATGCATAGATATCACGCAGAACTCCGATTGCGAAGGCAGCTTACTAAGGTACAACTGACGCTGAAGCACGAAAGCGTGGGTATCAAACAGGATTAGATAC																					
**ASV765**	GCTGCAGTGGGGAATCTTCCGCAATGGACGAAAGTCTGACGGAGCAACGCCGCGTGAGTGATGACGGCCTTCGGGTTGTAAAGCTCTGTTAATCGGGACGAATGGTTCTTGTGCGAATAGTGCGAGGATTTGACGGTACCGGAATAGAAAGCCACGGCTAACTACGTGCCAGCAGCCGCGGTAATACGTAGGTGGCAAGCGTTGTCCGGAATTATTGGGCGTAAAGCGCGCGCAGGCGGATTGGTCAGTCTGTCTTAAAAGTTCGGGGCTTAACCCCGTGATGGGATGGAAACTGCCAATCTAGAGTATCGGAGAGGAAAGTGGAATTCCTAGTGTAGCGGTGAAATGCGTAGATATTAGGAAGAACACCAGTGGCGAAGGCGACTTTCTGGACGAAAACTGACGCTGAGGCGCGAAAGCCAGGGGAGCGAACGGGATTAGATAC	0	0	0	0	0	0	0	0	0	0	573	701	499	163	186	290	400	25	474	197	95
**ASV773**	GCTGCAGTGAGGAATATTGGTCAATGGGCGAGAGCCTGAACCAGCCAAGTAGCGTGCAGGATG	0	0	0	0	0	0	0	0	0	0	429	644	745	0	0	85	369	0	279	97	0

	ACGGCCCTATGGGTTGTAAACTGCTTTTGTATGGGGATAAAGTCAATCACGTGTGATTGTTTGCAGGTACCATACGAATAAGGACCGGCTAATTCCGTGCCAGCAGCCGCGGTAATACGGAAGGTCCGGGCGTTATCCGGATTTATTGGGTTTAAAGGGAGCGTAGGCTGGAGATTAAGTGTGTTGTGAAATGTAGACGCTCAACGTCTGACTTGCAGCGCATACTGGTTTCCTTGAGTACGCACAACGTTGGCGGAATTCGTCGTGTAGCGGTGAAATGCTTAGATATGACGAAGAACTCCGATTGCGAAGGCAGCTGACGGGAGCGCAACTGACGCTGAAGCTCGAAGGTGCGGGTATCGAACAGGATTAGATAC																					
**ASV778**	GCTGCAGTGGGGAATCTTCCGCAATGGACGAAAGTCTGACGGAGCAACGCCGCGTGAGTGATGACGGCCTTCGGGTTGTAAAGCTCTGTTAATCGGGACGAAAGGTCCTCTTGCGAATAGTTAGAGGAATTGACGGTACCGGAATAGAAAGCCACGGCTAACTACGTGCCAGCAGCCGCGGTAATACGTAGGTGGCAAGCGTTGTCCGGAATTATTGGGCGTAAAGCGCGCGCAGGCGGATCAGTTAGTCTGTCTTAAAAGTTCGGGGCTTAACCCCGTGATGGGATGGAAACTGCTGATCTAGAGTATCGGAGAGGAAAGTGGAATTCCTAGTGTAGCGGTGAAATGCGTAGATATTAGGAAGAACACCAGTGGCGAAGGCGACTTTCTGGACGAAAACTGACGCTGAGGCGCGAAAGCCAGGGGAGCGAACGGGATTAGATAC	0	0	0	0	0	0	0	0	0	0	0	0	51	0	303	191	0	153	0	429	669

**ASV781**	GCTGCAGTGAGGAATATTGGTCAATGGACGGAAGTCTGAACCAGCCAAGTAGCGTGCAGGATGACGGCCCTATGGGTTGTAAACTGCTTTTGTATGGGGATAAAGTTAGGGACGTGTCCCTATTTGCAGGTACCATACGAATAAGGACCGGCTAATTCCGTGCCAGCAGCCGCGGTAATACGGAAGGTCCAGGCGTTATCCGGATTTATTGGGTTTAAAGGGAGCGTAGGCTGGAGATTAAGTGTGTTGTGAAATGTAGACGCTCAACGTCTGACTTGCAGCGCATACTGGTTTCCTTGAGTACGCACAACGTTGGCGGAATTCGTCGTGTAGCGGTGAAATGCTTAGATATGACGAAGAACTCCGATTGCGAAGGCAGCTGACGGGAGCGCAACTGACGCTTAAGCTCGAAGGTGCGGGTATCAAACAGGATTAGATAC	0	0	0	0	0	0	0	0	0	0	1322	28	39	278	0	0	7	15	14	0	25
**ASV787**	GCAGCAGTGGGGAATATTGCACAATGGGCGCAAGCCTGATGCAGCGACGCCGCGTGAGGGATGACGGCCTTCGGGTTGTAAACCTCTGTTAGCAGGGAAGAAGAGAGATTGACGGTACCTGCAGAGAAAGCGCCGGCTAACTACGTGCCAGCAGCCGCGGTAATACGTAGGGCGCGAGCGTTGTCCGGAATTATTGGGCGTAAAGAGCTTGTAGGCGGTTTGTCGCGTCTGCTGTGAAAGGCCGGAGCTTAACTCCGTGTATTGCAGTGGGTACGGGCAGACTAGAGTGCAGTAGGGGAGACTGGAATTCCTGGTGTAGCGGTGGAATGCGCAGATATCAGGAGGAACACCGATGGCGAAGGCAGGTCTCTGGGCTGTA	335	0	1519	940	281	872	1398	20	0	11131	0	0	0	91	0	199	0	220	136	219	0

	ACTGACGCTGAGAAGCGAAAGCATGGGGAGCGAACAGGATTAGATAC																					
**ASV792**	GCTGCAGTGAGGAATATTGGTCAATGGGCGAGAGCCTGAACCAGCCAAGTCGCGTGAAGGATGACTGTCTTATGGATTGTAAACTTCTTTTATACGGGAATAACAAGAGTCACGTGTGGCTCCCTGCATGTACCGTATGAATAAGCATCGGCTAACTCCGTGCCAGCAGCCGCGGTAATACGGAGGATGCGAGCGTTATCCGGATTTATTGGGTTTAAAGGGTGCGTAGGCGGCCTGTTAAGTCAGCGGTGAAATCTAGGAGCTTAACTCCTAAATTGCCATTGATACTGGCGGGCTTGAGTGTAGATGAGGTAGGCGGAATGCGTGGTGTAGCGGTGGAATGCATAGATATCACGCAGAACTCCGATTGCGAAGGCAGCTTACTAAGGTACAACTGACGCTGAAGCACGAAAGCGTGGGTATCAAACAGGATTAGATAC	0	0	0	0	0	0	0	0	0	0	0	0	36	0	422	51	211	92	0	323	307
**ASV795**	GCTGCAGTGGGGAATATTGCACAATGGGCGAAAGCCTGATGCAGCAACGCCGCGTGAACGATGAAGGTCTTCGGATCGTAAAGTTCTGTTGCAGGGGAAGATAATGACGGTACCCTGTGAGGAAGCCCCGGCTAACTACGTGCCAGCAGCCGCGGTAATACGTAGGGGGCTAGCGTTATCCGGATTTACTGGGCGTAAAGGGTGCGTAGGTGGTCCTTCAAGTCGGTGGTTAAAGGCTACGGCTCAACCGTAGTAAGCCGCCGAAACTGGAGGACTTGAGTGCAGGAGAGGAAAGTGGAATTCCCAGTGTAGCGGTGAAATGCGTAGATATTGGGAGGAACACCAG	0	0	0	0	0	0	0	0	0	0	0	0	0	398	140	92	100	798	0	349	682

	TAGCGAAGGCGGCTTTCTGGACTGCAACTGACACTGAGGCACGAAAGCGTGGGTAGCAAACAGGATTAGATAC																					
**ASV797**	GCTGCAGTAGGGAATCTTCGGCAATGGGGGCAACCCTGACCGAGCAACGCCGCGTGAGTGAAGAAGGTTTTCGGATCGTAAAGCTCTGTTGTAAGTCAAGAACGAGTGTGAGAGTGGAAAGTTCACACTGTGACGGTAGCTTACCAGAAAGGGACGGCTAACTACGTGCCAGCAGCCGCGGTAATACGTAGGTCCCGAGCGTTGTCCGGATTTATTGGGCGTAAAGCGAGCGCAGGCGGTTTGATAAGTCTGAAGTTAAAGGCTGTGGCTCAACCATAGTTCGCTTTGGAAACTGTCAAACTTGAGTGCAGAAGGGGAGAGTGGAATTCCATGTGTAGCGGTGAAATGCGTAGATATATGGAGGAACACCGGTGGCGAAAGCGGCTCTCTGGTCTGTAACTGACGCTGAGGCTCGAAAGCGTGGGGAGCGAACAGGATTAGATAC	0	0	0	0	0	0	0	0	0	0	72	223	1195	270	55	273	0	0	244	0	44
**ASV809**	GCTGCAGTGAGGAATATTGGTCAATGGGCGAGAGCCTGAACCAGCCAAGTAGCGTGCAGGATGACGGCCCTATGGGTTGTAAACTGCTTTTATGTGGGGATAAAGTGAGCTACGTGTAGTTTATTGCAGGTACCACATGAATAAGGACCGGCTAATTCCGTGCCAGCAGCCGCGGTAATACGGAAGGTCCAGGCGTTATCCGGATTTATTGGGTTTAAAGGGAGCGTAGGCCGTGGATTAAGCGTGTTGTGAAATGTAGACGCTCAACGTCTGAATTGCAGCGCGAA	0	0	0	0	0	0	0	0	0	0	92	424	193	55	78	0	162	0	174	88	75

	CTGGTTCACTTGAGTATGCACAACGTAGGCGGAATTCGTCGTGTAGCGGTGAAATGCTTAGATATGACGAAGAACTCCGATTGCGAAGGCAGCTTACGGGAGCACAACTGACGCTGAAGCTCGAAGGTGCGGGTATCAAACAGGATTAGATAC																					
**ASV814**	GCAGCAGTCGGGAATATTGCACAATGGAGGAAACTCTGATGCAGTGACACCGCGTATAGGAAGAAGGTCTTAGGATTGTAAGCTATTGTCGTGTGAGAAGAAAATGACCATCACAGGAGGAAGCCCTGGCTAAATATGTGCCAGCAGCCGCGGTAATACATATGGGGCGAGCGTTATCCGGATTTATTGGGTGTAAAGGGTGCGTAGGCGGGAAAATAAGTCAGTATGTGAAATCCCTCGGCTCAACTGAGGAACTGCAACTGAAACTATTTTTCTTGAGTGTTGAAGGGGAAAGTGGAATTCCTAGTGTAGCGGTGAAATGCGTAGAGATTAGGAGGAACACCAGTGGCGAAGGCGACTTTCTGGGCAACAACTGACGCTGAGGCACGAAAGTGTGGGGAGCAAACAGGATTAGATAC	148	0	16	72	0	80	30	0	0	0	0	92	0	35	203	267	356	51	20	229	92
**ASV816**	GCTGCAGTGAGGAATATTGGTCAATGGGCGCGAGCCTGAACCAGCCAAGTAGCGTGCAGGATGACGGCCCTATGGGTTGTAAACTGCTTTTGTATGGGGATAAAGTCAATCACGTGTGATTGTTTGCAGGTACCATACGAATAAGGACCGGCTAATTCCGTGCCAGCAGCCGCGGTAATACGGAAGGTCCGGGCGTTATCCGGATTTATTGGGTTTAAAGGGAGCGTAGGCCGGAGATTAAGTGTGTTGTGAAAT	0	0	0	0	0	0	0	0	0	0	510	238	185	0	41	230	0	0	103	0	26

	GTAGACGCTCAACGTCTGACTTGCAGCGCATACTGGTTTCCTTGAGTACGCACAACGTTGGCGGAATTCGTCGTGTAGCGGTGAAATGCTTAGATATGACGAAGAACTCCGATTGCGAAGGCAGCTGACGGGAGCGCAACTGACGCTTAAGCTCGAAGGTGCGGGTATCGAACAGGATTAGATAC																					
**ASV821**	GCTGCAGTGGGGAATATTGCACAATGGGGGAAACCCTGATGCAGCGACGCCGCGTGAGTGAAGAAGTATTTCGGTATGTAAAGCTCTATCAGCAGGGAAGATAATGACAGTACCTGACTAAGAAGCCCCGGCTAACTACGTGCCAGCAGCCGCGGTAATACGTAGGGGGCAAGCGTTATCCGGATTTACTGGGTGTAAAGGGAGCGTAGACGGAATGGCAAGTCTGAAGTGAAATACCCGGGCTCAACCTGGGAACTGCTTTGGAAACTGTTGTTCTAGAGTGTTGGAGAGGTAAGTGGAATTCCTGGTGTAGCGGTGAAATGCGTAGATATCAGGAAGAACACCGGAGGCGAAGGCGGCTTACTGGACAATAACTGACGTTGAGGCTCGAAAGCGTGGGGATCAAACAGGATTAGATAC	0	0	0	0	0	0	0	0	0	0	0	0	79	82	54	52	84	75	0	152	397
**ASV834**	GCTGCAGTGGGGAATATTGCACAATGGGCGCAAGCCTGATGCAGCAACGCCGCGTGAAGGATGAAGGCCTTCGGGTTGTAAACTTCTGTCCTAGGGGAAGAAACAAATGACATTACCCTTGGAGGAAGCCCCGGCTAACTACGTGCCAGCAGCCGCGGTAATACGTAGGGGGCGAGCGTTATCCGGAATTATTGGGCGTAAAGAGTGCGTAGGTGGCAC	0	0	0	0	0	0	0	0	0	0	0	0	123	76	0	171	331	103	0	115	304

	CTTAAGCGCAGGGTTTAAGGCAATGGCTCAACCATTGTTCGCCTTGCGAACTGGGGTGCTTGAGTGCAGGAGGGGAAAGTGGAATTCCTAGTGTAGCGGTGAAATGCGTAGATATTAGGAGGAACACCAGTGGCGAAGGCGACTTTCTGGACTGTTACTGACACTGAGGCACGAAAGCGTGGGGAGCAAACAGGATTAGATAC																					
**ASV842**	GCTGCAGTGAGGAATATTGGTCAATGGATGCAAATCTGAACCAGCCAAGTAGCGTGCAGGATGACGGCCCTATGGGTTGTAAACTGCTTTTATGTGAGAATAAAGTTAGGTATGTATACTTATTTGCATGTATCACATGAATAAGGACCGGCTAATTCCGTGCCAGCAGCCGCGGTAATACGGAAGGTCCAGGCGTTATCCGGATTTATTGGGTTTAAAGGGTGCGTAGGCCGTTTGATAAGCGTGCTGTGAAATATAGTGGCTCAACCTCTATCGTGCAGCGCGAACTGTTGAACTTGAGTGCGTAGTAGGTAGGCGGAATTCGTGGTGTAGCGGTGAAATGCTTAGATATCACGAAGAACTCCGATTGCGAAGGCAGCTTACCGTAACGTTACTGACGCTTAAGCACGAAGGTGCGGGTATCGAACAGGATTAGATAC	0	0	0	0	0	0	0	0	0	0	0	0	0	66	132	0	0	0	0	44	604
**ASV852**	GCTGCAGTAGGGAATCTTCCGCAATGGGCGAAAGCCTGACGGAGCAACGCCGCGTGAGTGAAGAAGGATTTCGGTTCGTAAAGCTCTGTTGTTAGGGAAGAATGATTGTATAGTAACTATATACAGTAGAGACGGTACCTAACCAGAAAGCCACGGCTAACTACGTGCCAGCAGCCGCGGTAATACGTAGG	0	0	0	0	0	0	0	0	0	0	110	0	44	41	61	151	34	0	27	69	141

	TGGCAAGCGTTGTCCGGAATTATTGGGCGTAA<br>AGCGCGCGCAGGTGGTTTAATAAGTCTGATGT<br>GAAAGCCCACGGCTCAACCGTGGAGGGTCATT<br>GGAAACTGTTAAACTTGAGTGCAGGAGAGAA<br>AAGTGGAATTCCTAGTGTAGCGGTGAAATGCG<br>TAGAGATTAGGAGGAACACCAGTGGCGAAGG<br>CGGCTTTTTGGCCTGTAACTGACACTGAGGCG<br>CGAAAGCGTGGGGAGCAAACAGGATTAGATA<br>C																					
**ASV855**	GCTGCAGTGAGGAATATTGGTCAATGGGCGAG<br>AGCCTGAACCAGCCAAGTCGCGTGAAGGATG<br>ACTGTCTTATGGATTGTAAACTTCTTTTATACG<br>GGAATAACAAGAGTCACGAGTGACTCCCTGC<br>ATGTACCGTATGAATAAGCATCGGCTAACTCC<br>GTGCCAGCAGCCGCGGTAATACGGAGGATGC<br>GAGCGTTATCCGGATTTATTGGGTTTAAAGGG<br>TGCGTAGGCGGCCTGTTAAGTCAGCGGTGAAA<br>TCTAGGAGCTTAACTCCTAAATTGCCATTGATA<br>CTGGCGGGCTTGAGTGTAGATGAGGTAGGCGG<br>AATGCGTGGTGTAGCGGTGGAATGCATAGATA<br>TCACGCAGAACTCCGATTGCGAAGGCAGCTTA<br>CTAAGGTACAACTGACGCTGAAGCACGAAAG<br>CGTGGGTATCAAACAGGATTAGATAC	0	0	0	0	0	0	0	0	0	0	0	0	0	0	588	40	42	0	29	65	351
**ASV857**	GCAGCAGTGAGGAATATTGGTCAATGGGCGA<br>GAGCCTGAACCAGCCAAGTAGCGTGCAGGAT<br>GACGGCCCTCCGGGTTGTAAACTGCTTTTAGTT<br>GGGAATAAAAAAAGGGACGTGTCCCTTCTTGT	0	0	0	0	0	0	0	0	0	0	0	0	203	0	38	1315	64	0	0	158	0

	ATGTACCTTCAGAAAAAGGACCGGCTAATTCCGTGCCAGCAGCCGCGGTAATACGGAAGGTCCAGGCGTTATCCGGATTTATTGGGTTTAAAGGGAGCGTAGGCGGATTGTTAAGTCAGCGGTTAAAGGGTGTGGCTCAACCATGCATTGCCGTTGAAACTGGCGATCTTGAGTGCAGACAGGGATGCCGGAATTCGTGGTGTAGCGGTGAAATGCTTAGATATCACGAAGAACTCCGATCGCGAAGGCAGGTGTCCGGGCTGCAACTGACGCTGAGGCTCGAAAGTGTGGGTATCAAACAGGATTAGATAC																					
**ASV859**	GCTGCAGTGAGGAATATTGGTCAATGGATGGAAATCTGAACCAGCCAAGTAGCGTGCAGGATGACGGCCCTATGGGTTGTAAACTGCTTTTATGTGAGAATAAAGTTAGGTATGTATACTTATTTGCATGTATCACATGAATAAGGACCGGCTAATTCCGTGCCAGCAGCCGCGGTAATACGGAAGGTCCAGGCGTTATCCGGATTTATTGGGTTTAAAGGGTGCGTAGGCCGTTTGATAAGCGTGCTGTGAAATATAGTGGCTCAACCTCTATCGTGCAGCGCGAACTGTCGAACTTGAGTGCGTAGTAGGTAGGCGGAATTCGTGGTGTAGCGGTGAAATGCTTAGATATCACGAAGAACTCCGATTGCGAAGGCAGCTTACCGTAACGTTACTGACGCTTAAGCACGAAGGTGCGGGTATCGAACAGGATTAGATAC	0	0	0	0	0	0	0	0	0	0	0	0	0	0	515	21	68	0	73	40	80
**ASV861**	GCAGCAGTGAGGAATATTGGACAATGGGTGGAAGCCTGATCCAGCCATCCCGCGTGCAGGACGACTGCCCTATGGGTTGTAAACTGCTTTTATATA	159	59	83	0	237	125	48	46	64	489	23	0	23	62	93	66	52	20	62	48	39

	GGGATAAACCTACTCTCGTGAGAGTAGCTGAAGGTACTATATGAATAAGCACCGGCTAACTCCGTGCCAGCAGCCGCGGTAATACGGAGGGTGCAAGCGTTATCCGGATTTATTGGGTTTAAAGGGTCCGTAGGTGGGCTGATAAGTCAGCGGTGAAATCCTGCAGCTTAACTGTAGAACTGCCGTTGATACTGTTAGTCTTGAGTGTATTTGAAGTGGCTGGAATAAGTAGTGTAGCGGTGAAATGCATAGATATTACTTAGAACACCAATTGCGAAGGCAGGTCACTAAGATACAACTGACGCTGAGGGACGAAAGCGTGGGGAGCGAACAGGATTAGATAC																					
**ASV865**	GCTGCAGTGGGGAATCTTGCACAATGGACGAAAGTCTGATGCAGCAATTTCGCGTGAAGGATGAAGCATTACGGTGTGTAAACTTCTTTTTTGGCAGAAGACGAATGACGGTATGTCAAGAATAAGAGACGGCTAACTACGTGCCAGCAGCCGCGGTAATACGTAGGTCTCAAGCGTTGTCCGGATTTACTGGGCGTAAAGTGTCCGTAGTCTGAATTGTAAGTCTGTTTTCAAATCCTACGACTCAATCGTAGAAAGGGAGTGGATACTGCAATTCTGGAAGTATCTGGGGGTTAGTGGAATTTCCGGTGGAGCGGTGAAATGCGTTGATATCGGAAGGAACGCCGAAAGCGAAAGCAGCTAACTACAGAATACTTGACGATGAGGGACGACAGTTCGGGTAGCAAACAGGATTAGATAC	0	0	0	0	0	0	0	0	0	0	0	0	0	56	38	60	0	61	0	261	361
**ASV875**	GCTGCAGTGGGGAATATTGGACAATGGAGGAGACTCTGATCCAGCAATTCTGTGTGCACGAAG	0	0	0	0	0	0	0	0	0	0	0	0	61	0	18	175	134	0	0	0	703

	ACGGTTTTCGGATTGTAAAGTGCTTTCAGCAGGGAAGAAGAAAGTGACGGTACCTGCAGAAGAAGCGACGGCTAAATACGTGCCAGCAGCCGCGGTAATACGTATGTCGCGAGCGTTATCCGGAATTATTGGGCATAAAGGGCATCTAGGCGGATATACAAGTCAGGGGTGAAAACTTAGGGCTCAACTCAAAGCTTGCCTTTGAAACTGTATATCTAGAGTGCTGGAGAGGTGGACGGAACTACACGAGTAGAGGTGAAATTCGTAGATATGTGTAGGAATGCCGATGATGAAGATAGTCCACTGGACAGCAACTGACGCTGAAGTGCGAAAGCTAGGGGAGCAAACAGGATTAGATAC																					
**ASV878**	GCTGCAGTAGGGAATATTGCTCAATGGGGGAAACCCTGAAGCAGCAACGCCGCGTGGAGGATGACACTTTTCGGAGCGTAAACTCCTTTTGTTAGGGAAGAACAATGACGGTACCTAACGAATAAGCACCGGCTAACTCCGTGCCAGCAGCCGCGGTAATACGGAGGGTGCAAGCGTTACTCGGAATCACTGGGCGTAAAGGACGCGTAGGCGGATTATCAAGTCTCTTGTGAAATCCTATGGCTTAACCATAGAACTGCTTGGGAAACTGATAATCTAGAGTGAGGGAGAGGCAGATGGAATTGGTGGTGTAGGGGTAAAATCCGTAGAGATCACCAGGAATACCCATTGCGAAGGCGATCTGCTGGAACTCAACTGACGCTAATGCGTGAAAGCGTGGGGAGCAAACAGGATTAGATAC	0	0	0	0	0	0	0	0	0	0	0	89	74	37	38	61	37	0	92	61	114

**ASV883**	GCTGCAGTGGGGAATATTGGACAATGGGGGAAACCCTGATCCAGCGACGCCGCGTGAGTGAAGAAGTATTTCGGTATGTAAAGCTCTATCAGCAGGGAAGAAAATGACGGTACCTGACTAAGAAGCCCCGGCTAACTACGTGCCAGCAGCCGCGGTAATACGTAGGGGGCAAGCGTTATCCGGATTTACTGGGTGTAAAGGGAGCGCAGACGGCAATGCAAGTCTGAAGTGAAAGGCGTGGGCTCAACCCATGAACTGCTTTGGAAACTGTATAGCTTGAGTGTCGGAGGGGTAAGCGGAATTCCTAGTGTAGCGGTGAAATGCGTAGATATTAGGAGGAACACCGGAGGCGAAGGCGGCTTACTGGACGACAACTGACGTTGAGGCTCGAAGGCGTGGGGAGCAAACAGGATTAGATAC	0	0	0	0	0	0	0	0	0	0	0	0	41	0	0	114	94	65	0	86	153
**ASV893**	GCTGCAGTGGGGAATATTGCACAATGGGGGAAACCCTGATGCAGCGACGCCGCGTGAGTGAAGAAGTATTTCGGTATGTAAAGCTCTATCAGCAGGGAAGATAATGACAGTACCTGACTAAGAAGCCCCGGCTAACTACGTGCCAGCAGCCGCGGTAATACGTAGGGGGCAAGCGTTATCCGGATTTACTGGGTGTAAAGGGAGCGTAGACGGAATGGCAAGTCTGAAGTGAAAACCCCGGGCTCAACCTGGGGACTGCTTTGGAAACTGTTGTTCTAGAGTGTTGGAGAGGTAAGTGGAATTCCTGGTGTAGCGGTGAAATGCGTAGATATCAGGAAGAACACCGGAGGCGAAGGCGGCTTACTGGACAATAACTGA	0	0	0	0	0	0	0	0	0	0	0	0	30	0	111	131	0	51	0	58	183

	CGTTGAGGCTCGAAAGCGTGGGGATCAAACAGGATTAGATAC																					
**ASV894**	GCTGCAGTAGGGAATCTTCCGCAATGGACGCAAGTCTGACGGAGCAACGCCGCGTGAGTGAAGAAGGATTTCGGTTCGTAAAACTCTGTTGTTAGAGAAGAACAGCGCATAGAGTAACTGTTATGCGTGTGACGGTATCTAACCAGAAAGCCACGGCTAACTACGTGCCAGCAGCCGCGGTAATACGTAGGTGGCAAGCGTTGTCCGGATTTATTGGGCGTAAAGCGAGCGCAGGCGGTCAATTAAGTCTGATGTGAAAGCCCCCGGCTCAACCGGGGAGGGTCATTGGAAACTGGTTGACTTGAGTGCAGAAGAGGAGAGTGGAATTCCATGTGTAGCGGTGAAATGCGTAGATATATGGAGGAACACCAGTGGCGAAGGCGACTCTCTGGTCTGTAACTGACGCTGAGGCTCGAAAGCGTGGGTAGCAAACAGGATTAGATAC	0	0	0	0	0	0	0	0	0	0	6	56	0	0	0	0	0	0	147	19	0
**ASV896**	GCTGCAGTGGGGAATATTGCACAATGGGGGAAACCCTGATGCAGCGACGCCGCGTGAGCGAAGAAGGTTTTCGAATCGTAAAGCTCTGTCCTATGAGAAGATAATGACGGTATCATAGGAGGAAGCCCCGGCTAAATACGTGCCAGCAGCCGCGGTAATACGTATGGGGCGAGCGTTGTCCGGAATTATTGGGCGTAAAGGGTACGTAGGCGGCCTTTTAAGTCAGGTGTGAAAGCGTGAGGCTTAACCTCATTAAGCACTTGAAACTGGAAGGCTTGAGTGAAGGAGAGGAAAGTGGAATTCCTAGTGTAGCGG	0	0	0	0	0	0	0	0	0	0	0	0	0	0	173	0	29	0	0	100	94

	TGAAATGCGTAGATATTAGGAGGAATACCGGTGGCGAAGGCGACTTTCTGGACTTTTACTGACGCTCAGGTACGAAAGCGTGGGGAGCAAACAGGATTAGATAC																					
**ASV901**	GCTGCAGTGGGGAATATTGCACAATGGGCGCAAGCCTGATGCAGCGACGCCGCGTGAGGGATGACGGCCTTCGGGTTGTAAACCTCTGTTAGCATCGAAGAAGCGAAAGTGACGGTAGGTGCAGAGAAAGCGCCGGCTAACTACGTGCCAGCAGCCGCGGTAATACGTAGGGCGCGAGCGTTGTCCGGAATTATTGGGCGTAAAGAGCTTGTAGGCGGTTGGTCGCGTCTGCTGTGAAAGGCTGGGGCTTAACCCTGGTTTTGCAGTGGGTACGGGCTAACTAGAGTGCAGTAGGGGAGACTGGAATTCCTGGTGTAGCGGTGGAATGCGCAGATATCAGGAGGAACACCGATGGCGAAGGCAGGTCTCTGGGCTGTAACTGACGCTGAGAAGCGAAAGCATGGGGAGCGAACAGGATTAGATAC	0	0	0	0	0	0	0	0	0	0	99	0	42	0	55	0	0	0	180	23	21
**ASV905**	GCTGCAGTCGGGAATATTGCACAATGGAGGAAACTCTGATGCAGTGACACCGCGTATAGGAAGAAGGTCTTAGGATTGTAAGCTATTGTCGTGTGAGAAGAAAATGACCATCACAGGAGGAAGCCCTGGCTAAATATGTGCCAGCAGCCGCGGTAATACATATGGGGCGAGCGTTATCCGGATTTATTGGGTGTAAAGGGTGCGTAGGCGGGAAAATAAGTCAGTATGTGAAATCCCTCGGCTCAACTGAGGAACTGCAACTGAAACTATTTTTCTTGAGTGTTGA	0	0	0	0	0	0	0	0	0	0	0	37	0	0	74	92	139	0	0	95	99

	AGGGGAAAGTGGAATTCCTAGTGTAGCGGTG AAATGCGTAGAGATTAGGAGGAACACCAGTG GCGAAGGCGACTTTCTGGGCAACAACTGACGC TGAGGCACGAAAGTGTGGGGAGCAAACAGGA TTAGATAC																				
**ASV906**	GCTGCAGTGGGGAATATTGGACAATGGGGGC AACCCTGATCCAGCAATTCTGTGTGCACGAAG ACGGTTTTCGGATTGTAAAGTGCTTTCAGCAG GGAAGAAGGAAGTGACGGTACCTGCAGAAGA AGCGACGGCTAAATACGTGCCAGCAGCCGCG GTAATACGTATGTCGCGAGCGTTATCCGGAAT TATTGGGCATAAAGGGCATCTAGGCGGCCTTT CAAGTCAGGGGTGAAAACCTGCGGCTCAACC GCAGGCCTGCCTTTGAAACTGATAGGCTGGAG TACCGGAGAGGTGGACGGAACTGCACGAGTA GAGGTGAAATTCGTAGATATGTGCAGGAATGC CGATGATGAAGATAGTTCACTGGACGGTAACT GACGCTGAAGTGCGAAAGCCGGGGGAGCGAA CAGGATTAGATAC	0	0	0	0	0	0	0	0	0	0	0	0	0	0	128	104	214	0	0	20	117
**ASV920**	GCTGCAGTAGGGAATCTTCGGCAATGGGGGG AACCCTGACCGAGCAACGCCGCGTGAGTGAA GAAGGTTTTCGGATCGTAAAGCTCTGTTGTAA GAGAAGAACGGGTGTGAGAGTGGAAAGTTCA CACTGTGACGGTATCTTACCAGAAAGGGACGG CTAACTACGTGCCAGCAGCCGCGGTAATACGT AGGTCCCGAGCGTTGTCCGGATTTATTGGGCG TAAAGCGAGCGCAGGCGGTTAGATAAGTCTG	0	0	0	0	0	0	0	0	0	0	0	0	0	45	81	82	43	0	28	111	38

	AAGTTAAAGGCTGTGGCTTAACCATAGTATGCTTTGGAAACTGTTTAACTTGAGTGCAGAAGGGGAGAGTGGAATTCCATGTGTAGCGGTGAAATGCGTAGATATATGGAGGAACACCGGTGGCGAAAGCGGCTCTCTGGTCTGTAACTGACGCTGAGGCTCGAAAGCGTGGGGAGCAAACAGGATTAGATAC																					
**ASV964**	GCAGCAGTGAGGAATATTGGTCAATGGGCGCGAGCCTGAACCAGCCAAGTAGCGTGCAGGATGACGGCCCTATGGGTTGTAAACTGCTTTTGTATGGGGATAAAGTTAGGGACGTGTCCCTATTTGCAGGTACCATACGAATAAGGACCGGCTAATTCCGTGCCAGCAGCCGCGGTAATACGGAAGGTCCAGGCGTTATCCGGATTTATTGGGTTTAAAGGGAGCGTAGGCTGGAGATTAAGTGTGTTGTGAAATGTAGACGCTCAACGTCTGAATTGCAGCGCATACTGGTTTCCTTGAGTACGCACAACGTTGGCGGAATTCGTCGTGTAGCGGTGAAATGCTTAGATATGACGAAGAACTCCGATTGCGAAGGCAGCTGACGGGAGCGCAACTGACGCTTAAGCTCGAAGGTGCGGGTATCAAACAGGATTAGATAC	0	0	0	0	0	0	0	0	0	0	0	129	0	0	0	380	0	0	26	0	38
**ASV967**	GCTGCAGTGGGGAATATTGCACAATGGGGGAAACCCTGATGCAGCGACGCCGCGTGAGTGAAGAAGTATTTCGGTATGTAAAGCTCTATCAGCAGGGAAGAAAATGACGGTACCTGAGTAAGAAGCCCCGGCTAACTACGTGCCAGCAGCCGCGGTAATACGTAGGGGGCAAGCGTTATCCGGATTTAC	0	0	0	0	0	0	0	0	0	0	234	54	79	0	0	0	0	0	79	0	0

	TGGGTGTAAAGGGAGCGCAGACGGCTCTGCA<br>AGTCTGAAGTGAAAGCCCGCGGCTTAACCGCG<br>GGACTGCTTTGGAAACTGCAAGGCTTGAGTAT<br>CGGAGGGGCAGGCGGAATTCCTAGTGTAGCG<br>GTGAAATGCGTAGATATTAGGAAGAACACCG<br>GTGGCGAAGGCGGCCTGCTGGACGAAAACTG<br>ACGTTGAGGCTCGAAGGCGTGGGGAGCAAAC<br>AGGATTAGATAC																					
**ASV979**	GCTGCAGTGGGGGATATTGCACAATGGAGGA<br>AACTCTGATGCAGCGACGCCGCGTGAGTGAA<br>GAAGTATTTCGGTATGTAAAGCTCTATCAGCA<br>GGGAAGATGATGACGGTACCTGACTAAGAAG<br>CCCCGGCTAACTACGTGCCAGCAGCCGCGGTA<br>ATACGTAGGGGGCAAGCGTTATCCGGATTTAC<br>TGGGTGTAAAGGGAGCGCAGGCGGTTTGGCA<br>AGTTGAGAGTGGAAGCAGGGGGCTCAACCCC<br>CTGACTGCTCCCAAAACTGTTAAACTTGAGTA<br>TGGGAGAGGCAGGCGGAATTCCTAGTGTAGC<br>GGTGAAATGCTTAGATATTAGGAAGAACACC<br>GGTGGCGAAGGCGGCCTGCTGGACCAAAACT<br>GACGCTGAGGCTCGAAAGCGTGGGTAGCAAA<br>CAGGATTAGATAC	0	0	0	0	0	0	0	0	0	0	0	0	0	0	39	0	43	0	0	79	105
**ASV1001**	GCTGCAGTGGGGAATATTGCACAATGGGGGA<br>AACCCTGATGCAGCGACGCCGCGTGAGTGAA<br>GAAGTATTTCGGTATGTAAAGCTCTATCAGCA<br>GGGAAGATAATGACAGTACCTGACTAAGAAG<br>CCCCGGCTAACTACGTGCCAGCAGCCGCGGTA	0	0	0	0	0	0	0	0	0	0	0	0	0	0	0	0	33	0	53	32	39

	ATACGTAGGGGGCAAGCGTTATCCGGATTTAC<br>TGGGTGTAAAGGGAGCGTAGACGGAGTGGCA<br>AGTCTGAAGTGAAAACCCTGGGCTTAACCTGG<br>GGACTGCTTTGGAAACTGTTAATCTAGAGTGTT<br>GGAGAGGTAAGTGGAATTCCTGGTGTAGCGGT<br>GAAATGCGTAGATATCAGGAAGAACACCGGA<br>GGCGAAGGCGGCTTACTGGACAATAACTGAC<br>GTTGAGGCTCGAAAGCGTGGGGATCAAACAG<br>GATTAGATAC																						
**ASV1010**	GCAGCAGTGGGGAATATTGGACAATGGGGGG<br>AACCCTGATCCAGCGACGCCGCGTGAGTGAA<br>GAAGTATTTCGGTATGTAAAGCTCTATCAGCA<br>GGGAAGAAAATGACGGTACCTGACTAAGAAG<br>CCCCGGCTAACTACGTGCCAGCAGCCGCGGTA<br>ATACGTAGGGGGCAAGCGTTATCCGGATTTAC<br>TGGGTGTAAAGGGAGCGCAGACGGCTTGGCA<br>AGTCTGAAGTGAAAGCCCGGGGCTTAACCCCG<br>GGACTGCTTTGGAAACTGTGAAGCTAGAGTGT<br>CGGAGAGGTAAGTGGAATTCCCAGTGTAGCG<br>GTGAAATGCGTAGATATTGGGAGGAACACCG<br>GTGGCGAAGGCGGCTTACTGGACGATAACTG<br>ACGTTGAGGCTCGAAGGCGTGGGGAGCAAAC<br>AGGATTAGATAC	0	0	43	0	0	0	23	0	0	0	0	0	21	39	17	14	65	43	0	34	24
**ASV1015**	GCAGCAGCTAAGAATATTCCGCAATGGACGG<br>AAGTCTGACGGAGCGACGCCGCGTGGATGAA<br>GAAGGCTGAAAAGTTGTAAAATCCTTTTGTTG<br>ATGAAGAATAAGGGTGAGAGGGAATGCTCAT	0	0	0	0	0	0	0	0	0	0	0	0	0	0	46	52	11	0	0	89	17

	CTGATGACGGTAATCGACGAATAAGCCCCGGCTAATTACGTGCCAGCAGCCGCGGTAACACGTAAGGGGCGAGCGTTGTTCGGAATTATTGGGCGTAAAGGGCATGTAGGCGGTTGTGTAAGCCTGGTGTGAAATCCTGGGGCTTAACCCCAGAATAGCATTGGGTACTGCACGACTTGAATTACGGAAGGGAAACTGGAATTCCAAGTGTAGGGGTGGAATCTGTAGATATTTGGAAGAACACCGGTGGCGAAGGCGGGTTTCTGGCCGATAATTGACGCTGAGATGCGAAAGTGTGGGGATCGAACAGGATTAGATAC																					
**ASV1050**	GCAGCAGTGGGGGATATTGCACAATGGGGGGAACCCTGATGCAGCAACGCCGCGTGAGTGATGAAGGCCCTAGGGTCGTAAAGCTCTGTCGTATGGGAAGAAACAAATGACTGTACCATACAAGAAAGCCCCGGCTAACTACGTGCCAGCAGCCGCGGTAATACGTAGGGGGCAAGCGTTATCCGGAATTACTGGGCGTAAAGGGTGCGTAGGCGGCTAATTAAGTCGAGGTTAAAAGGCAGTAGCTCAACTATTGTTGGGCCTTGAAACTAATTAGCTTGAGTATAGGAGAGGAAAGTGGAATTCCCGGTGTAGCGGTGAAATGCGTAGATATCGGGAGGAATACCGGTGGCGAAGGCGACTTTCTGGACTATAACTGACGCTGAGGCACGAAAGCGTGGGTAGCAAACAGGATTAGATAC	0	0	0	0	0	0	0	0	0	0	0	0	0	0	0	0	17	4	0	315	49
**ASV1101**	GCTGCAGTGAGGAATATTGGACAATGGTCGGAAGACTGATCCAGCCATGCCGCGTGCAGGAAG	0	0	0	0	0	0	0	0	0	0	0	0	0	26	40	0	0	0	0	83	28

	ACGGCCTTATGGGTTGTAAACTGCTTTTGCAGGGGAAGAATAAGGAGTACGTGTACTTTGATGACGGTACTCTGCGAATAAGCATCGGCTAACTCCGTGCCAGCAGCCGCGGTAATACGGAGGATGCGAGCGTTATCCGGAATCATTGGGTTTAAAGGGTCCGTAGGCGGGCTAATAAGTCAGAGGTGAAAGCGCTCAGCTCAACTGAGCAACTGCCTTTGAAACTGTTAGTCTTGAATGGTTGTGAAGTAGTTGGAATGTGTAGTGTAGCGGTGAAATGCTTAGATATTACACAGAACACCGATAGCGAAGGCATATTACTAACAATTAATTGACGCTGATGGACGAAAGCGTGGGGAGCGAACAGGATTAGATAC																					
**ASV1153**	GCTGCAGTAGGGAATATTGCGCAATGGGGGAAACCCTGACGCAGCAACGCCGCGTGGAGGATGACACTTTTCGGAGCGTAAACTCCTTTTGTTAGGGAAGAATAATGACGGTACCTAACGAATAAGCACCGGCTAACTCCGTGCCAGCAGCCGCGGTAATACGGAGGGTGCAAGCGTTACTCGGAATCACTGGGCGTAAAGGACGCGTAGGCGGATTATCAAGTCTCTTGTGAAATCTAGTGGCTTAACCACTAAACTGCTTGGGAAACTGATAATCTAGAGTAAGGGAGAGGCAGATGGAATTCTTGGTGTAGGGGTAAAATCCGTAGAGATCAAGAAGAATACTTATTGCGAAGGCGATCTGCTAGAACTTAACTGACGCTAATGCGTGAAAGCGTGGGGAGCAAACAGGATTAGATAC	0	0	0	0	0	0	0	0	0	0	0	0	0	171	0	0	47	0	5	0	32

**ASV1158**	GCTGCAGTGAGGAATATTGGACAATGGTCGGAAGACTGATCCAGCCATGCCGCGTGCAGGATGAAGGTCCTATGGATTGTAAACTGCTTTTGTAAGGGAAGAATAAGGAGTACGTGTACTTTGATGACGGTACCTTACGAATAAGCATCGGCTAACTCCGTGCCAGCAGCCGCGGTAATACGGAGGATGCGAGCGTTATCCGGAATCATTGGGTTTAAAGGGTCCGTAGGCGGGCTGATAAGTCAGAGGTGAAAGCGCTTAGCTCAACTAAGCAACTGCCTTTGAAACTGTCAGTCTTGAATGATTGTGAAGTAGTTGGAATGTGTAGTGTAGCGGTGAAATGCTTAGATATTACACAGAACACCGATAGCGAAGGCATATTACTAACAATTTATTGACGCTGATGGACGAAAGCGTGGGGAGCGAACAGGATTAGATAC	0	0	0	0	0	0	0	0	0	0	0	0	0	0	36	0	0	14	0	155	20
**ASV1161**	GCTGCAGTGGGGAATCTTCCGCAATGGGCGCAAGCCTGACGGAGCAACGCCGCGTGAGTGAAGAAGGTCTTCGGATCGTAAAGCTCTGTTGACGGGGACGAACGTGCGGGGTGCGAATAGCGCTTTGTAATGACGGTACCTGTCGAGGAAGCCACGGCTAACTACGTGCCAGCAGCCGCGGTAATACGTAGGTGGCGAGCGTTGTCCGGAATCATTGGGCGTAAAGGGAGCGCAGGCGGGCCGGTAAGTCTTACTTAAAAGTGCGGGGCTCAACCCCGTGATGGGAGAGAAACTATCGGTCTTGAGTACAGGAGAGGAAAGCGGAATTCCCAGTGTAGCGGTGAAATGCGTAGATATTGGGAAGAACACCAGTGGCGAAGGCGGCTTTCTGGACTGCAACTGACGCTGAG	0	0	0	0	0	0	0	0	0	0	0	23	34	0	68	0	38	0	0	0	0

	GCTCGAAAGCCAGGGGAGCGAACGGGATTAGATAC																					
**ASV1329**	GCAGCAGTAGGGAATCTTCGGCAATGGGGGGAACCCTGACCGAGCAACGCCGCGTGAGTGAAGAAGGTTTTCGGATCGTAAAGCTCTGTTGTAAGAGAAGAACGGGTGTGAGAGTGGAAAGTTCACACTGTGACGGTATCTTACCAGAAAGGGACGGCTAACTACGTGCCAGCAGCCGCGGTAATACGTAGGTCCCGAGCGTTGTCCGGATTTATTGGGCGTAAAGCGAGCGCAGGCGGTTAGATAAGTCTGAAGTTAAAGGCTGTGGCTTAACCATAGTACGCTTTGGAAACTGTTTAACTTGAGTGCAGAAGGGGAGAGTGGAATTCCATGTGTAGCGGTGAAATGCGTAGATATATGGAGGAACACCGGTGGCGAAAGCGGCTCTCTGGTCTGTAACTGACGCTGAGGCTCGAAAGCGTGGGGAGCAAACAGGATTAGATAC	0	0	0	0	0	0	0	0	0	0	0	42	0	1	3	7	2	35	2	0	0
**ASV1363**	GCAGCAGTGAGGAATATTGGTCAATGGGCGAGAGCCTGAACCAGCCAAGTAGCGTGCAGGATGACGGCCCTCCGGGTTGTAAACTGCTTTTAGTTGGGAATAAAAAAAGGGACTTGTCCCTTCTTGTATGTACCTTCAGAAAAAGGACCGGCTAATTCCGTGCCAGCAGCCGCGGTAATACGGAAGGTCCAGGCGTTATCCGGATTTATTGGGTTTAAAGGGAGCGTAGGCGGATTGTTAAGTCAGCGGTTAAAGGGTGTGGCTCAACCATACATTGCCGTTGAAACTGGCGATCTTGAGTGCAGACAGGGATGCCGG	0	0	0	0	0	0	0	0	0	0	0	0	0	14	4	0	1	0	0	0	2

	AATTCGTGGTGTAGCGGTGAAATGCTTAGATATCACGAAGAACTCCGATCGCGAAGGCAGGTGTCCGGGCTGCAACTGACGCTGAGGCTCGAAAGTGTGGGTATCAAACAGGATTAGATAC																					
**ASV1405**	GCAGCAGCTAAGAATCTTCCGCAATGGGCGCAAGCCTGACGGAGCGACGCCGCGTGAATGAAGAAGGCCGAAAGGTTGTAAAATTCTTTTAAGTTTGAAGAATAACCAGTATAGGGAATGATACTGAGATGACGGTAAAACTTGAATAAGCCCCGGCTAATTACGTGCCAGCAGCCGCGGTAACACGTAAGGGGCGAGCGTTGTTCGGAATTATTGGGCGTAAAGGGTATGCAGGCGGTTAGATAAGCCCGGTGTGAAATGCTCAAGCTTAACTTGAGAACTGCACTAGGTACTGTCTAACTTGAATCACGAAGGGGAAACTGGAATTCCAGGTGTAGGGGTGGAATCTGTAGATATCTGGAAGAATACCGGTGGCGAAGGCGGGTTTCTGGTCGATGATTGACGCTGAGATACGAAAGTGTGGGGAGCAAACAGGATTAGATAC	0	0	0	0	0	0	0	0	0	0	0	0	0	0	7	0	0	10	0	40	13
**ASV1414**	GCAGCAGTAGGGAATCTTCGGCAATGGACGAAAGTCTGACCGAGCAACGCCGCGTGAGTGAAGAAGGTTTTCGGATCGTAAAGCTCTGTTGTAAGAGAAGAACGGGTGTGAGAGTGGAAAGTTCACACTGTGACGGTATCTTACCAGAAAGGGACGGCTAACTACGTGCCAGCAGCCGCGGTAATACGTAGGTCCCGAGCGTTGTCCGGATTTATTGGGCGTAAAGCGAGCGCAGGCGGTTAGATAAGTCTG	0	0	0	0	0	0	0	0	0	0	1	2	23	37	0	0	0	0	2	0	0

	AAGTTAAAGGCTGTGGCTTAACCATAGTACGCTTTGGAAACTGTTTAACTTGAGTGCAGAAGGGGAGAGTGGAATTCCATGTGTAGCGGTGAAATGCGTAGATATATGGAGGAACACCGGTGGCGAAAGCGGCTCTCTGGTCTGTAACTGACGCTGAGGCTCGAAAGCGTGGGGAGCAAACAGGATTAGATAC																					
**ASV1473**	GCAGCAGTGGGGGATATTGCACAATGGAGGAAACTCTGATGCAGCGACGCCGCGTGAGTGAAGAAGTATTTCGGTATGTAAAGCTCTATCAGCAGGGAAGAAAGACTCGAAAGAGAGATGACGGTACCTGACTAAGAAGCTCCGGCTAAATACGTGCCAGCAGCCGCGGTAATACGTATGGAGCAAGCGTTATCCGGATTTACTGGGTGTAAAGGGAGCGCAGACGGTTATGCAAGTCTGAAGTGAAAAACCACGGCTCAACTGTGGTCTTGCTTTGGAAACTGTGTAACTAGAGTGTCGGAAGGGTAAGCGGAATTCCTAGTGTAGCGGTGAAATGCGTAGATATTAGGAAGAACACCGGAGGCGAAGGCGGCTTACTGGACGATAACTGACGTTGAGGCTCGAAGGCGTGGGTAGCAAACAGGATTAGATAC	0	0	0	0	0	0	0	0	0	0	0	0	6	69	0	0	0	0	3	0	13
**ASV1569**	GCTGCAGTAGGGAATCTTTCACAATGGGCGAAAGCCTGATGGAGCAACGCCGCGTGCAGGATGAAGGCCTTCGGGTTGTAAACTGCTTTTATAAGCGAGAAATATGATGGTAACTTATGAATAAGGATCGGCTAACTACGTGCCAGCAGCCGCGGTCATACGTAGGATCCGAGCATTATCCGGAGTGACT	0	0	0	0	0	0	0	0	0	0	0	0	0	0	18	0	8	0	0	20	37

	GGGTGTAAAGAGTTGCGTAGGTGGCATAGTAA<br>GTAGATAGTGAAATCTGGTGGCTCAACCATTC<br>AGACTATTATCTAAACTGCTAAGCTCGAGACC<br>GTTAGGGGTAACTGGAATTTCTAGTGTAGGAG<br>TGAAATCCGTAGATATTAGAAGGAACACCGAT<br>AGCGTAGGCAGGTTACTGGGACGGTTCTGACA<br>CTAAGGCACGAAAGCGTAGGGAGCAAACGGG<br>ATTAGATAC																					
**ASV1600**	GCAGCAGTGGGGAATATTGGGCAATGGGAGG<br>AATCCTGACCCAGCGACGCCGCGTGAACGAA<br>GACGGCCTTCGGGTTGTAAAGTTCTTTTATGTG<br>GGAAGAAGGAAGTGACGGTACCACATGAATA<br>AGCCCCGGCTAACTACGTGCCAGCAGCCGCG<br>GTAATACGTAGGGGGCGAGCGTTGTCCGGAAT<br>TACTGGGCGTAAAGGGCACGCAGGCTGTGCTT<br>CAAGTCAGCTGTAAAAGGATGCGGCTTAACCG<br>TGTTATGCAGTTGAGACTGAGGTGCTGGAGTA<br>CCGGAGAGGCAAGTGGAATTCCCAGTGTAGC<br>GGTGAAATGCGTAGATATTGGGAAGAACATC<br>GGTGGCGAAGGCGACTTGCTGGACGGTAACTG<br>ACGCTGAGGTGCGAAAGCCAGGGTAGCGAAC<br>GGGATTAGATAC	0	0	0	0	0	0	0	0	0	0	0	4	5	39	0	0	0	25	0	0	0

p-value<0.05; #*p-value*<0.1.

Table S4. Description of the sequencing run and processing

Sample Code	Group	Input reads	Quality-filtered reads	Denoised reads Forward	Denoised reads Reverse	Paired-end merged reads	Non-chimeric reads	Final reads
**56**	Control	79908	62444	61829	62032	60519	54601	54601
**58**	Control	102243	78502	77419	77831	75389	71334	71334
**60**	Control	99651	71118	69912	70317	67779	64016	64016
**65**	Control	124833	91466	90088	90755	87173	82955	82955
**67**	Control	115307	84464	82861	83501	79513	76139	76139
**69**	Control	92486	67008	65412	66120	62136	59336	59336
**73**	Control	105805	80748	79740	80065	77069	70409	70409
**75**	Control	108758	81360	80240	80733	78720	76665	76665
**77**	Control	91382	67852	66682	67191	64834	63685	63685
**82**	Control	97737	72821	72294	72476	71252	68432	68432
**85**	Control	89930	65055	63228	64049	60371	57376	57376
**116**	UC	149803	116075	114284	114992	110618	101948	101948
**118**	UC	141359	111029	109818	110277	106798	93466	93466
**120**	UC	136138	96031	94148	94952	90306	82309	82309
**122**	UC	109042	84824	84002	84063	82794	78065	78065
**124**	UC	103869	75887	75533	75616	74393	70769	70769
**126**	UC	118484	87416	86804	87006	85449	83263	83263
**128**	UC	152860	115980	113633	114896	109248	99097	99097
**130**	UC	136590	104676	103532	103926	100432	93799	93799
**132**	UC	131378	93634	92659	93269	91037	80523	80523
**134**	UC	116715	86682	85498	86083	82568	75494	75494

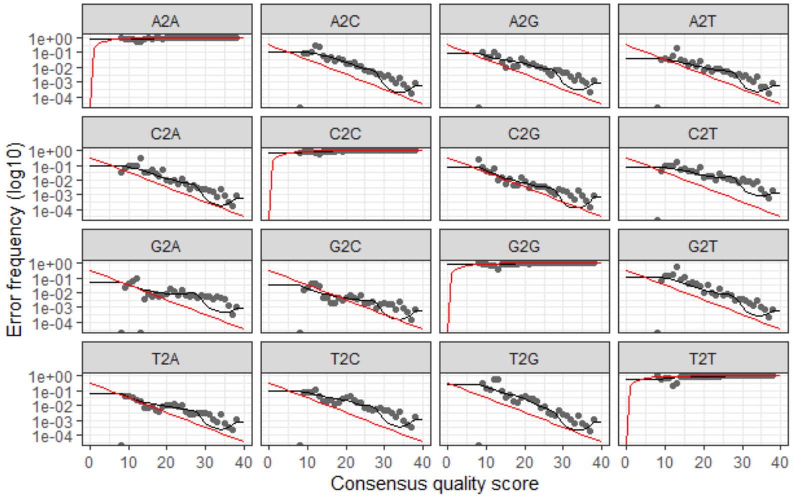

Figure S1. Plot showing error rates for each possible transition (A→C, A→G, …) (Points are the observed error rates for each consensus quality score). The black line shows the estimated error rates after convergence of the machine-learning algorithm. The red line shows the error rates expected under the nominal definition of the Q-score. Here the estimated error rates (black line) are a good fit to the observed rates (points), and the error rates drop with increased quality as expected.

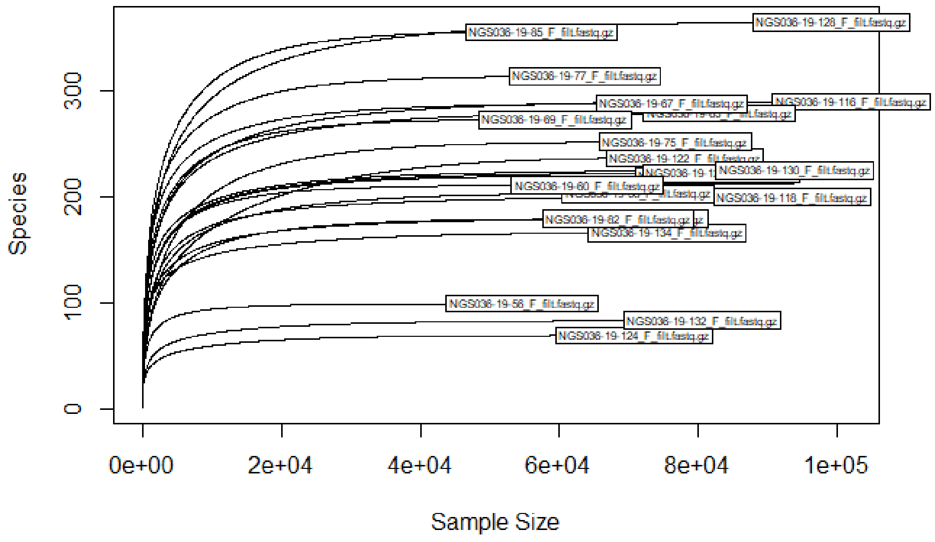

Figure S2. Rarefaction curves per sample that show the sequencing depth of the study.
